# Meal timing of dietary total antioxidant capacity and its association with all-cause, CVD and cancer mortality: the US national health and nutrition examination survey, 1999–2018

**DOI:** 10.1186/s12966-023-01487-1

**Published:** 2023-07-07

**Authors:** Peng Wang, Xuye Jiang, Qilong Tan, Shanshan Du, Dan Shi

**Affiliations:** 1 Department of Nutrition and Food Hygiene, School of Public Health, Chongqing Medical University, Chongqing, China; 2 Department of Epidemiology and Biostatistics, School of Public Health, Fujian Medical University, Fuzhou, Fujian China; 3 Foundation Centre for Basic Metabolic Research, Faculty of Health and Medical Sciences, University of Copenhagen, Copenhagen, Denmark; 4 Department of Epidemiology and Biostatistics, School of Public Health, Zhejiang University School of Medicine, Hangzhou, Zhejiang China; 5 Research Centre for Environment and Human Health, School of Public Health, Chongqing Medical University, Chongqing, China; 6 Nutrition Innovation Platform-Sichuan and Chongqing, School of Public Health, Chongqing Medical University, Chongqing, China

**Keywords:** DAC, Mortality, Meal timing, NHANES

## Abstract

**Background:**

The association of the meal timing of dietary total antioxidant capacity (DAC) with mortality is unclear. We aimed to investigate the association between the meal timing of DAC and all-cause, cardiovascular disease (CVD), and cancer mortality in general adult populations.

**Methods:**

A total of 56,066 adults who participated in the US National Health and Nutrition Examination Survey (NHANES) from 1999 to 2018 were recruited for this study. Dietary intake (quantity and timing) was evaluated by nonconsecutive 24-h dietary recalls. The main exposure variables were the DAC across three meals (total, breakfast, lunch, and dinner; without coffee) and the difference between dinner and breakfast DAC (Δ = dinner-breakfast; without coffee). The outcomes were all-cause, CVD, and cancer mortality. The adjusted hazard ratios [aHRs] and 95% confidence intervals [CI] were imputed by Cox proportional hazards regression.

**Results:**

Among the 56,066 participants, there were 8566 deaths from any cause, including 2196 from CVD and 1984 from cancer causes. Compared to participants in the lowest quintiles of the total DAC, those in the highest quintiles had 34% and 27% decreased risks of all-cause and CVD mortality, respectively (all-cause mortality: aHRs 0.66 [95% CI 0.57–0.76]; CVD mortality: aHRs 0.73 [95% CI 0.57–0.94]). More importantly, participants in the highest quintiles of the dinner DAC, but not those in that of breakfast or lunch, had a 24% decrease in all-cause mortality (aHRs 0.76 [95% CI 0.67–0.87]) compared with those in the lowest quintiles. Inverse associations were further confirmed for Δ DAC (aHRs 0.84 [95% CI 0.74–0.96]). Above associations did not change when including DAC from snacks or tea. Mediation analysis showed that the total associations of total, dinner or Δ DACs with reduced all-cause mortality were 24%, 13% and 6%, respectively, mediated by serum CRP. Additionally, all-cause mortality was decreased by 7% in models replacing 10% breakfast DAC (aHRs 0.93 [95% CI 0.9–0.97]) with an equivalent proportion of dinner DAC. For cancer mortality, no statistical significance was detected in the adjusted models.

**Conclusions:**

The findings emphasize the putative beneficial relationship of a diet rich in antioxidants and meal timing on serum CRP and all-cause mortality.

**Supplementary Information:**

The online version contains supplementary material available at 10.1186/s12966-023-01487-1.

## Introduction

Dietary antioxidants, as potential inflammation and oxidative stress inhibitors, have attracted our attention in the field of nutrition. Health benefits of individual dietary antioxidants have been extensively reported, such as a reduced risk of all-cause, CVD and cancer mortality [1–3]. However, some controversial reports have revealed that single dietary antioxidant supplementation is not associated with oxidative stress-related diseases (such as cancer or CVD) and is sometimes even harmful [4, 5]. The inconsistent results appear to address that individual compounds are not sufficient to combat oxidative stress-related diseases; thus, study of combined dietary total antioxidant capacity (DAC) is warranted when considering the cumulative/synergistic effects of multiple dietary antioxidants in foods [6, 7].

Recently, DAC, as a composite measure of individual antioxidants, has been used to investigate the health effects of antioxidants present in mixed diets. Total DAC has been inversely related to cancer, diabetes, and other chronic diseases [6–11]. A small number of studies have also emerged to investigate the associations between total DAC and all-cause, cancer or CVD mortality risk in various contexts, among which four studies have reported an inverse relationship in adults [12–15], but one has reported a nonsignificant association with all-cause mortality in elderly individuals at high cardiovascular risk [16], which may be due to different age characteristics. Among these studies, results from the French E3N cohort or Singapore Chinese Health Study showed that total DAC was inversely associated with death from all-causes, cancer, or CVD in general adults [12, 14]. Another two studies from large NHANES data also revealed that in general individuals or adults with diabetes, overall dietary antioxidants were associated with a lower risk of all-cause and/or CVD mortality [13, 15]. Nevertheless, no study is available examining the association of meal timing of DAC with all cause, cancer, and CVD mortality.

Accumulating evidence has revealed that, in addition to the quantity and quality of food, meal timing is also crucial for the well-being of the organism [17]. More specifically, disruption of meal timing is associated with metabolic disorders and increased risk of chronic diseases [18, 19]. Abundant studies have also identified the optimal intake times of energy, macronutrients, vitamins, or minerals across a day for reducing the risk of all-cause, CVD, or cancer mortality [20–23]. Meal timing, as a potential zeitgeber, aids organisms in synchronizing the external environment to the internal circadian rhythm, thereby maximizing health improvement [24, 25]. Therefore, we hypothesized that the meal timing of DAC would also impact long-term health, especially all-cause, CVD and cancer mortality.

To test this hypothesis, in this study, we derived individual DAC values from the Antioxidant Food Database based on investigation from the NHANES. This study aimed to evaluate the association between meal timing of DAC and all-cause, CVD and cancer mortality risk in general adult populations from the 1999–2018 NHANES cohort data.

## Materials and methods

The reporting followed the Strengthening the Reporting of Observational Studies in Epidemiology (STROBE) guideline [26] (STROBE-nut guidelines; Additional file 1).

### Study Population

**Fig. 1:**
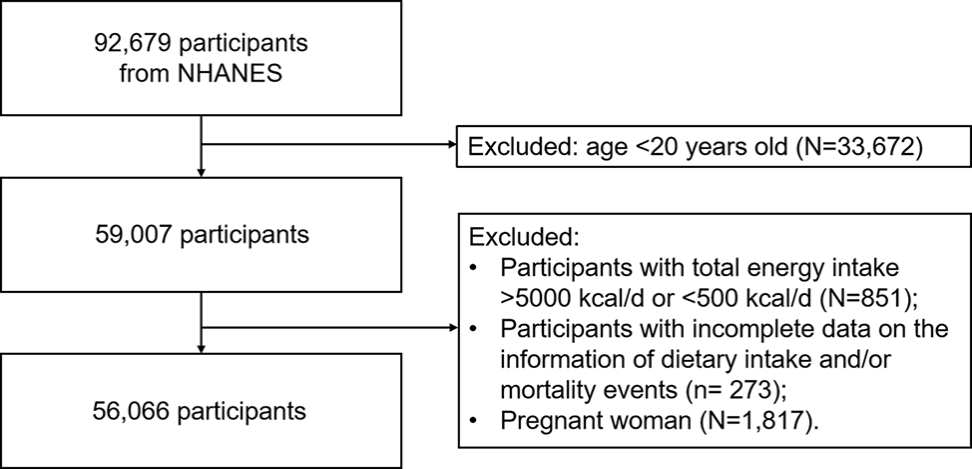
Cohort flow diagram in NHANES 1999–2018

The involved study populations were derived from the NHANES public dataset, a large prospective cohort on the health and nutritional data of the US population. NHANES was approved by the research ethics review board of the National Centre for Health Statistics Research. All participants provided informed consent at enrolment, and detailed information has been described elsewhere [27]. A total of 92,679 participants who finished at least one valid dietary recall of the NHANES from 1999 to 2018 were included at baseline. Participants aged < 20 years (n = 33,672), with a total energy intake > 5000 kcal/d or < 500 kcal/d (n = 851), who were pregnant (n = 1,817) and who lacked complete information on dietary intake and/or mortality events (n = 273) were excluded from the study. The final sample used in the current study comprised 56,066 participants (Fig. 1).

### Dietary Assessment

Baseline dietary information (quantity and timing) was gathered. For 13.5% of the population’s dietary information, dietary intake data from 1999 to 2002 were evaluated by one 24-h dietary recall interview; the rest of dietary intake from 2003 to 2018 was evaluated by two nonconsecutive 24-h dietary recall interviews. The first was conducted in person by well-trained staff at NHANES mobile examination centres, and the second was conducted exactly 3–10 days later over the telephone. The release of 2 days of data permits the estimation of usual (long-run average) nutrient intakes to assess diets in the U.S., which has been effectively validated [28]. Standardized protocols and measuring tools were applied to facilitate the assessment of the food volume and dimensions during the interview. The main three meals (breakfast, lunch, dinner) or snack events were directly reported from the interviews. The average food intake from 2003 to 2018 during the 2 days was calculated [29]. Nutrient values were calculated with reference to the Food and Nutrient Database for Dietary Studies of United States Department of Agriculture. Dietary supplements were acquired by a dietary supplement questionnaire [30]. The snack, coffee, or tea timing was set as 4:00 am-11:00 am for breakfast, 11:00 am-4:00 pm for lunch and 4:00 pm-10:00 pm for dinner [29].

### Dietary antioxidant capacity calculation

The main exposure variables of this study were the DAC from three main meals (total, breakfast, lunch, and dinner) and the difference between dinner DAC and breakfast DAC (Δ = dinner-breakfast). ‘Total’ indicated the sum of the three main meals of a day. The DAC was determined using the ferric-reducing ability of plasma (FRAP) assay, which measures the reduction of ferric ion (Fe3+) to ferrous ion (Fe2+) [31]. Then, the DAC was calculated by energy adjustment (nutrient residual model) [32] according to the Antioxidant Food Database, which is publicly available online on Oslo’s University website. The Antioxidant Food Database contains the antioxidant content of over 3100 types of processed or unprocessed ingredients from distinct foods, ranging from typical foods, traditional medicine plants, herbs, spices to dietary supplements [33]. Although the Antioxidant Food Database provides sufficient data to cover the antioxidant values of most dietary ingredients, antioxidant contents of several foods are still not available, including 27 fruits, 8 grains, 97 vegetables, 86 protein foods, 27 dairy products, 6 oils, 9 solid fats, and 7 added sugars. To address this, we used the antioxidant value of a similar item to fill in the antioxidant information of foods based on similarities, or we used the mean antioxidant values of the uniform group of foods if foods do not have similar substitutes (Additional file 2: Supplementary Table 1). We computed the dietary antioxidant capacity of each meal for each participant by multiplying the serving size for each food consumption in each meal by the FRAP value of that food obtained from the Antioxidant Food Database and then summed up across different food categories defined by the USDA’s Food Patterns Equivalents Database 2015–2016 for each individual. To minimize the effects of data distribution on our analysis, the mean and median DAC values of the food components were both calculated (Additional file 2: Supplementary Table 2). We excluded the DAC data derived from dietary supplements considering the missing data of intake time. Coffee is highly rich in antioxidants, which may blur the associations between DAC from other foods and mortality risk [7]. Therefore, coffee DAC was taken into account only in the sensitivity analysis but not in the main analysis in this study. Total, breakfast, lunch, and dinner DAC data were defined as the DAC without coffee in this study.

### Main outcome

The main outcomes were all-cause, CVD, and cancer mortality. The date of death and cause of death were verified through linkage with the National Death Index (NDI) public dataset. The International Classification of Diseases 10th Revision (ICD-10) codes were utilized to classify causes of death. Death from CVD was defined by the ICD-10 codes I00-I09, I11, I13, I20-I51, or I60-I69, and death from cancer was identified by the ICD-10 codes C00-C9. Overall, 8566 deaths from all causes were counted; of them, 2196 deaths were from CVD and 1984 deaths were from cancer.

### Assessment of Covariates

Baseline covariates were documented, including age, sex (male/female), race/ethnicity (Mexican American/non-Hispanic black/non-Hispanic white/other Hispanic/other), education (less than 9th grade/9-11th grade/college graduate or above/high school graduate (GED or equivalent)/some college or AA degree), family income ($0-$19,999/$20,000-$44,999/$45,000-$74,999/$75,000-$99,999/$100,000 and over), body mass index (BMI, kg/m^2^), alcohol intake (g/day), smoking status (never smoked/past smoker/current smoker), physical activity (metabolic equivalent score per week, METs-h/week), dietary energy intake (kcal), adherence to Healthy Eating Index 2015 (HEI-2015) score, dietary supplement use (%), serum C-reactive protein level (CRP, mg/dL), diabetes, hypertension, cancer, hyperlipidaemia, and CVD. Physical activity was acquired by Global Physical Activity Questionnaire. Diabetes was defined as haemoglobin A1c (HbA1c) ≥ 6.5%, or fasting plasma glucose ≥ 7.0 mmol/L, self-reported, or diagnosed diabetes [21]. Hypertension was defined as diagnosed hypertension, and hyperlipidaemia was defined as diagnosed hyperlipidaemia or taking antihyperlipidaemic drugs as reported in NHANES. HEI-2015 was derived to measure diet quality, a validated measurement score for assessing whether diets comply with the 2015 Dietary Guidelines for Americans [34].

### Statistical analyses

All analyses adopted the complex sampling method with sample weights, stratification, and clustering with reference to the NHANES analytic guidelines. Statistical analyses were performed by R version 4.2.0 (the R Core Team). A two-sided *P* ≤ 0.05 was considered statistically significant. Missing data were imputed using multivariate imputation by chained equations [35]. The results are presented as means, medians, means ± SEs, or means ± SDs for continuous variables or numbers (percentages) for categorical variables, as appropriate with weight adjustment. DAC across the day (total, breakfast, lunch, dinner) and the Δ DAC were divided into quintiles; quintile 1 was defined as the lowest DAC intake and quintile 5 as the highest. For baseline characteristics analysis, one-way ANOVA and the chi-square test of independence were used for continuous variables and categorical variables, respectively. Subgroup analysis was further performed, categorized by age, sex, race/ethnicity, education, income, BMI, smoking status, drinking, HEI-2015 score and physical activity in CPH models.

Survey-weighted Cox proportional hazards (CPH) models were applied to evaluate the associations between DAC (total, breakfast, lunch, dinner, and Δ; by quintiles) and all-cause, CVD, and cancer-related mortality. Adjusted hazard ratios (aHRs) and 95% confidence intervals (CIs) were computed. Three models adjusted for covariates were assessed. Model 1 was adjusted for age, sex, and race. Model 2 was additionally adjusted for education, family income, dietary energy intake, alcohol consumption per day, smoking status, physical activity, and BMI. Model 3 was further adjusted for diabetes, hypertension, CVD, cancer, hyperlipidaemia, adherence to the HEI-2015 score, and dietary supplement use. Models for breakfast, lunch, or dinner DACs were further adjusted except for the one that defined the DAC group.

### Substitution analysis

Next, to further evaluate the association of breakfast or dinner DAC with mortality risk in the general population, we carried out substitution model analysis to partition the risks of one dietary item into another to calculate the relative risk in a fixed amount of intake [21, 36]. Here, we evaluated the equivalent substitution relationship within the DAC group, wherein we estimated the aHRs by the replacement of 10% of DAC or its food sources at dinner for the equivalent amount of DAC at breakfast.

#### Regression-based causal mediation analysis

A regression-based causal mediation analysis was constructed to examine whether the association between total, dinner or Δ DACs and the risk of all-cause mortality was mediated by serum CRP with adjustment for covariates [37, 38]. Briefly, (1) a linear model (unweighted) for the association between total, dinner or Δ DAC and serum CRP level; (2) fully adjusted Cox models (unweighted) were conducted to measure the relationship DAC and serum CRP level on all-cause mortality; (3) these two above regression models were integrated for mediation analysis to evaluate the mediating role of serum CRP to DAC intake and all-cause mortality [39]. The total effect (TE) of total, dinner or Δ DACs on all-cause mortality decomposed into 2 components: the natural indirect effect (NIE) size and the natural direct effect (NDE) size. The proportion of the association between DAC and all-cause mortality mediated through CRP was calculated by the log (indirect association HR)/log (total association HR). Regression-based causal mediation analysis was performed using the R package regmedint [40].

### Sensitivity analyses

In sensitivity analysis, we assessed whether the relationship persisted after replacing the mean value of DAC with the median value in CPH models. Late-night eating is a significant risk factor for chronic diseases [19, 41]; thus, we further added midnight snack data and repeated the CPH analysis. Midnight snacking was defined as food consumption after 10 pm [42]. We also repeated the association between DAC and mortality using nonimputation data in weighted CPH. Moreover, we repeated the CPH analysis after the inclusion of DAC from snacks, coffee, and/or tea.

## Results

### Baseline characteristics

The baseline characteristics of all involved participants are shown in Table 1. This study included 56,066 adult participants with a mean age of 45.97 years; of them, 27,933 (49.8%) were women. The mean contents of total, breakfast, lunch, and dinner DACs (without coffee) were 4.03 mmol, 0.99 mmol, 1.84 mmol, and 1.20 mmol, respectively, with a Δ DAC value of 0.22. The mean contents of coffee DAC from total, breakfast, lunch, and dinner were 5.55 mmol, 4.01 mmol, 2.00 mmol, and 1.38 mmol, respectively. The mean contents of snack DAC from total, breakfast, lunch, and dinner were 1.85 mmol, 1.07 mmol, 2.31 mmol, and 1.86 mmol, respectively. The mean contents of tea DAC from total, breakfast, lunch, and dinner were 1.20 mmol, 0.27 mmol, 0.42 mmol, and 0.51 mmol, respectively. The detailed antioxidant capacity values of NHANES participants who consumed specific foods at breakfast, lunch, and dinner are further presented in Additional file 2: Supplementary Table 3. Approximately 33.1%, 30.4%, 16.9%, 16.0%, and 9.1% of individuals had a history of hypertension, CVD, hyperlipidaemia, diabetes, and cancer, respectively. The baseline characteristics of total, dinner, and Δ DACs value by quintiles are shown in Additional file 2: Supplementary Tables 4, 5 and 6. Participants in a higher total and dinner DACs quintiles were more likely to be older, adhere to HEI score, had lower BMI, had lower alcohol intake, had higher dietary supplement use, had less physical activity and were less likely to be current smokers, while participants with higher Δ DAC quintiles reported younger ages, had higher alcohol intake, had lower dietary supplement use, had lower adherence to HEI score and were less likely to have hypertension, diabetes, cancer, hyperlipidaemia, and CVD.

**Table 1:** Baseline characteristics of participation enrolled in the NHANES

Characteristic	Mean ± SE or n (%)
**Patients, n**	56,066
**Age (years)**	45.97 ± 0.20
**Female**	27,933 (49.8)
**Race/ethnicity**	
Mexican American	9314 (16.6)
Non-Hispanic Black	11,411 (20.4)
Non-Hispanic White	26,797 (47.8)
Other Hispanic	4149 (7.4)
Other	4395 (7.8)
**Education**	
Less than 9th grade	5784 (10.3)
9-11th grade	7577 (13.5)
College graduate or above	13,386 (23.9)
High school graduate/GED or equivalent	12,686 (22.7)
Some college or AA degree	16,575 (29.6)
**Income**	
$ 0 to $ 19,999	12,829 (22.9)
$20,000 to $44,999	18,656 (33.3)
$45,000 to $74,999	10,832 (19.3)
$75,000 to $99,999	9026 (16.1)
$100,000 and Over	4723 (8.4)
**Body mass index (kg/m** ^**2**^ **)**	28.42 ± 0.08
**Alcohol intake (g/day)**	10.02 ± 0.26
**Smoking status**	
Never smoked	30,509 (54.4)
Past smoker	14,074 (25.1)
Current smoker	11,483 (20.5)
**Physical activity (METs-h/week)**	11.11 ± 0.09
**Dietary energy intake (kcal)**	2136.20 ± 8.78
**Adherence to HEI-2015 score**	50.76 ± 0.19
**Total DAC intake (without coffee, mmol)**	4.03 ± 0.04
**Breakfast DAC intake (without coffee, mmol)**	0.99 ± 0.02
**Lunch DAC intake (without coffee, mmol)**	1.84 ± 0.03
**Dinner DAC intake (without coffee, mmol)**	1.20 ± 0.01
**Midnight DAC intake (without coffee, mmol)** ^**a**^	0.38 ± 0.01
**Δ DAC value (without coffee, mmol)** ^**b**^	0.22 ± 0.02
**Total Coffee DAC (mmol)**	5.55 ± 0.10
**Breakfast coffee DAC intake (mmol)**	4.01 ± 0.06
**Lunch coffee DAC intake (mmol) without coffee**,	2.00 ± 0.03
**Dinner coffee DAC intake (mmol)**	1.38 ± 0.02
**Δ coffee DAC value (mmol)** ^**b**^	-2.63 ± 0.06
**Total snack DAC (mmol)**	1.85 ± 0.03
**Breakfast snack DAC intake (mmol)**	1.07 ± 0.02
**(continued)**	
**Lunch snack DAC intake (mmol)**	2.31 ± 0.03
**Dinner snack DAC intake (mmol)**	1.86 ± 0.02
**Δ snack DAC value (mmol)** ^**b**^	0.79 ± 0.03
**Total tea DAC (mmol)**	1.20 ± 0.05
**Breakfast tea DAC intake (mmol)**	0.27 ± 0.01
**Lunch tea DAC intake (mmol)**	0.42 ± 0.02
**Dinner tea DAC intake (mmol)**	0.51 ± 0.03
**Δ tea DAC value (mmol)** ^**b**^	0.24 ± 0.03
**Dietary supplement use (%)**	33,046 (58.9)
**Serum CRP level (mg/dL)**	5.15 ± 0.14
**Hypertension**	18,585 (33.1)
**CVD**	17,050 (30.4)
**Hyperlipidaemia**	9483 (16.9)
**Diabetes**	8970 (16.0)
**Cancer**	5077 (9.1)

Abbreviations: BMI, body mass index; HEI-2015, Healthy Eating Index 2015; CVD, cardiovascular disease; DAC, dietary total antioxidant capacity; CRP, C-reactive protein; METs-h, metabolic equivalent scores

Continuous variables were adjusted for survey weights of NHANES. Categorical variables were unweighted

^a^ DAC intake from midnight snack after dinner; ^b^ Δ equals dinner DAC minus breakfast DAC.

### Cox Proportional Models

**Fig. 2:**
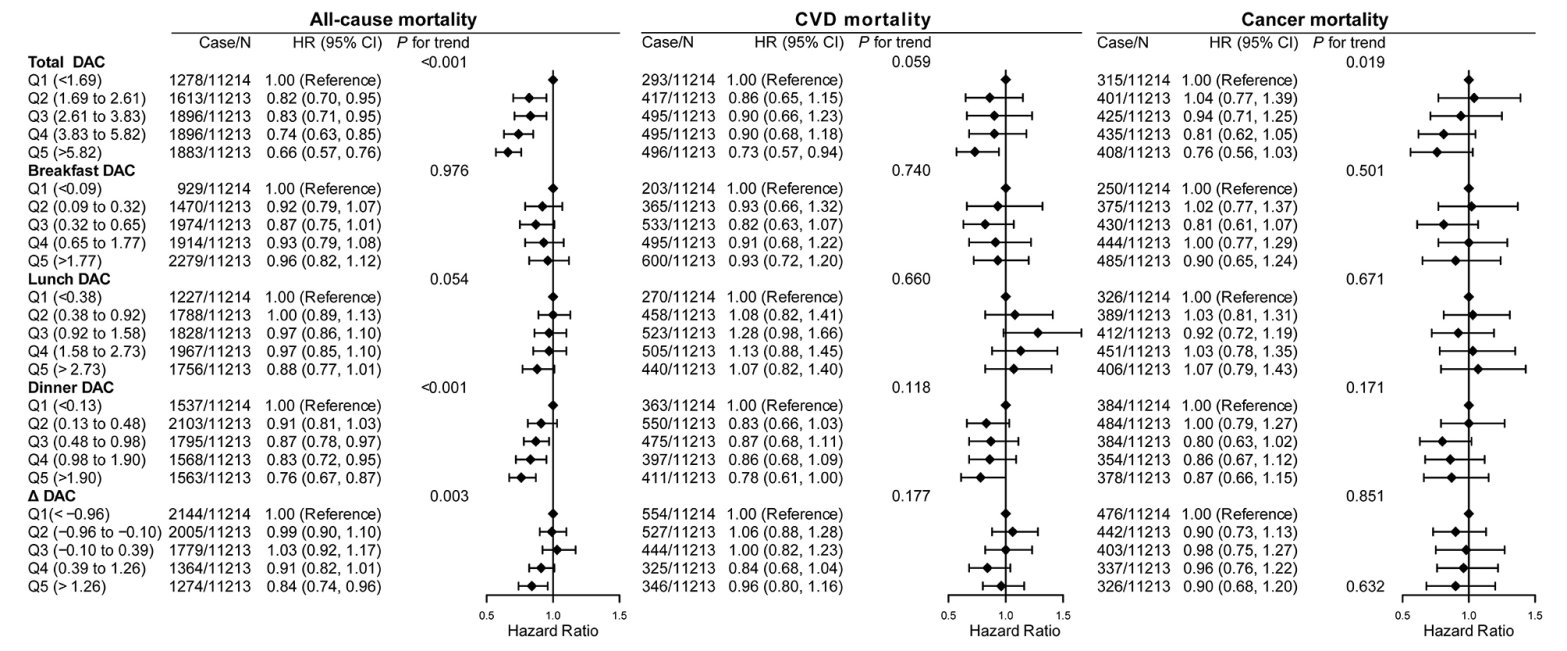
Associations of all-cause, CVD, and cancer mortality with quintiles of meal timing of DAC. Abbreviations: HRs, hazard ratio; CIs, confidence intervals; DAC, dietary total antioxidant capacity; HEI-2015, Healthy Eating Index 2015; BMI, body mass index; CVD, cardiovascular disease * *P* for trend across the quintile of DAC. HR (95%CI) was estimated by weighted Cox regression analyses. Δ equals dinner DAC minus breakfast DAC. Model was adjusted for, age, sex, race, education, family income, dietary energy intake, alcohol consumption per day, smoking status, physical activity, BMI, diabetes, hypertension, CVD, cancer, hyperlipidaemia, adherence to HEI-2015 score, and dietary supplement use Models for breakfast DAC, lunch DAC and dinner DAC were further adjusted except the one that defined the group

During a median follow-up of 12.75 years, 8566 of the 56,066 participants died, including 2196 CVD deaths and 1984 cancer-related deaths (Fig. 1). The association of DAC with mortality were evaluated (Fig. 2 and Additional file 2: Supplementary Tables 7, 8 and 9). Compared with quintiles 1 of total DAC, the aHRs and 95% CIs in quintiles 5 were 0.66 (0.57–0.76) for all-cause mortality (*P* for trend < 0.001), 0.73 (0.57–0.94) for CVD mortality (*P* for trend = 0.059), and 0.76 (0.56–1.03) for cancer mortality (*P* for trend = 0.019). All associations of DAC intake from breakfast or lunch with all-cause, CVD, and cancer mortality were nonsignificant after multivariable adjustment. In contrast, compared to the lowest quintile of dinner DAC, participants in the highest quintile were less likely to die due to all-cause mortality (aHRs 0.76 [95% CI 0.67–0.87], *P* for trend < 0.001) and CVD mortality (aHRs 0.78 [95% CI 0.61-1.00], *P* for trend = 0.118), but not cancer mortality. Likewise, significant associations were further found for Δ DAC with reduced all-cause mortality (aHRs 0.84 [95% CI 0.74–0.96], *P* for trend = 0.003) but not for CVD and cancer mortality.

**Fig. 3:**
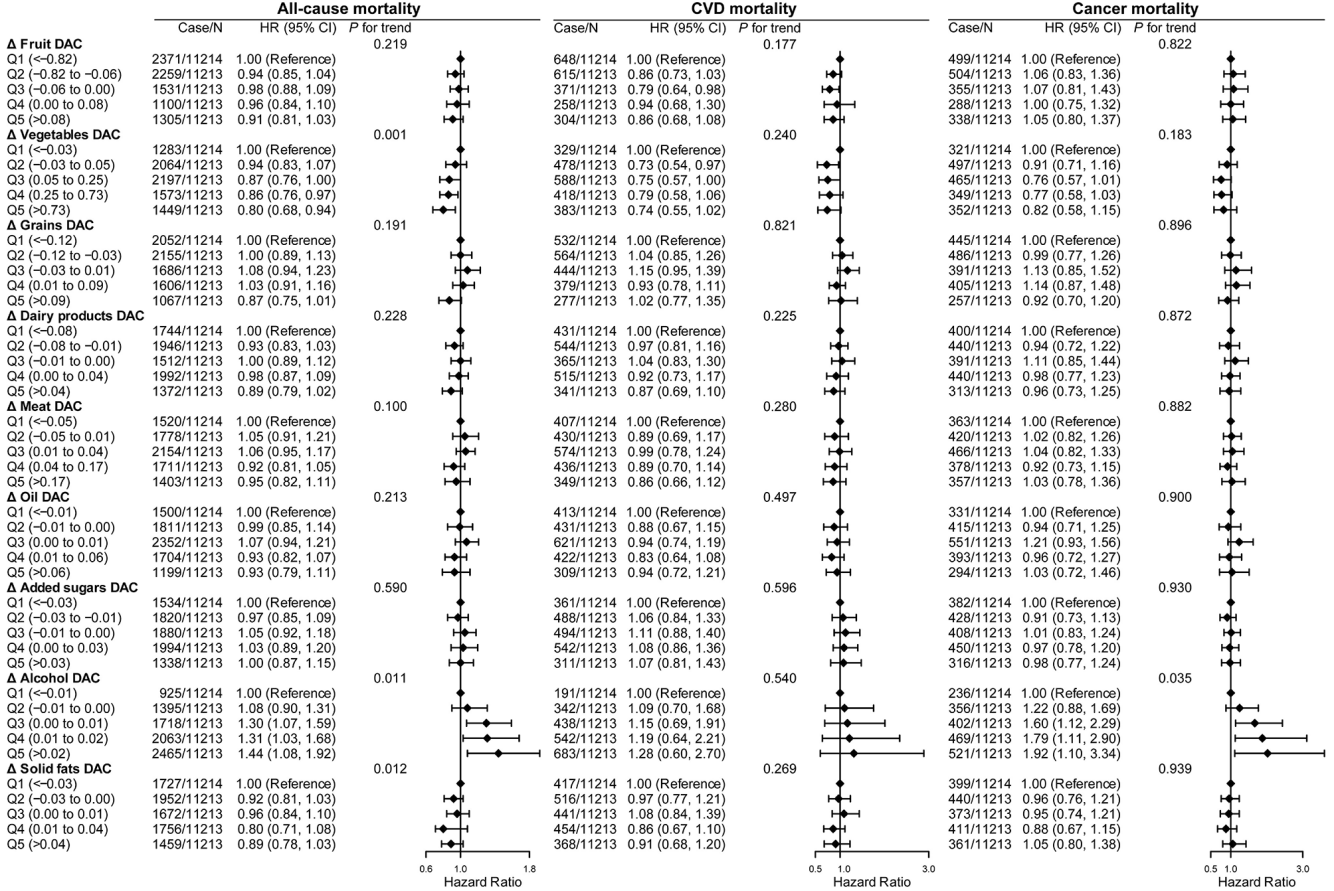
Adjusted HRs for Δ DAC components and all-cause, CVD, and cancer mortality Abbreviations: HRs, hazard ratio; CIs, confidence intervals; DAC, dietary total antioxidant capacity; HEI-2015, Healthy Eating Index 2015; BMI, body mass index; CVD, cardiovascular disease * *P* for trend across the quintile of DAC. HR (95%CI) was estimated by weighted Cox regression analyses. Δ equals dinner DAC components minus breakfast DAC components Model was adjusted for, age, sex, race, education, family income, dietary energy intake, alcohol consumption per day, smoking status, physical activity, BMI, diabetes, hypertension, CVD, cancer, hyperlipidaemia, adherence to HEI-2015 score, and dietary supplement use

Furthermore, the association of a given Δ DAC category defined by food components with all cause, CVD, and cancer mortality is shown in Fig. 3. Compared to the lowest quintile, participants in the highest quintile of Δ vegetables DAC had a lower risk of all-cause mortality (aHRs 0.80 [95% CI 0.68–0.94], *P* for trend = 0.001), while participants in the highest quintile of Δ alcohol DAC had greater all-cause (aHRs 1.44 [95% CI 1.08–1.92], *P* for trend = 0.011) and cancer mortality (aHRs 1.92 [95% CI 1.10–3.34], *P* for trend = 0.035). However, Δ DAC from other foods, including fruit, grains, dairy products, meat, oil, added sugars and solid fats, was not related to all-cause, CVD or cancer mortality.

### Subgroup analysis

Furthermore, subgroup analysis revealed that sex, race/ethnicity, education, BMI, drinking, HEI-2015 score and physical activity did not impact the association between total, dinner and Δ DACs with all-cause mortality (Additional file 2: Supplementary Tables 10, 11 and 12). However, inverse associations between total and dinner DACs with all-cause mortality were significantly observed in smokers rather than non-smokers (total DAC: *P* for interaction < 0.001; dinner DAC: *P* for interaction = 0.027). Higher dinner DAC was related to a lower all-cause mortality risk in those aged < 65 years rather than aged ≥ 65 years (*P* for interaction < 0.001). A significant interaction between income and Δ DAC was also observed.

### Substitution analysis

**Fig. 4:**
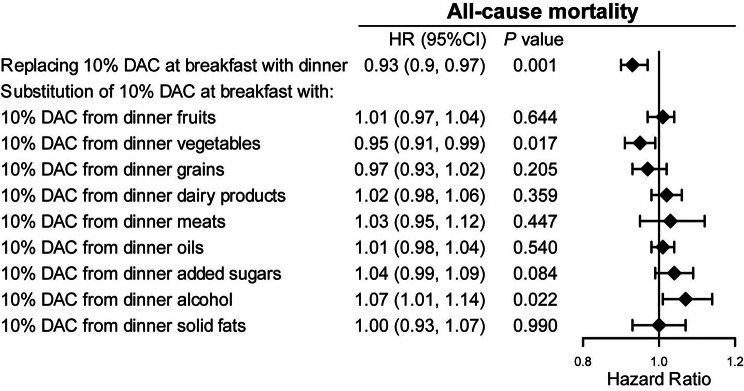
HRs with 95% CIs for all-cause mortality: substitution model of DAC at breakfast with dinner Abbreviations: HRs, hazard ratio; CIs, confidence intervals; DAC, dietary total antioxidant capacity; HEI-2015, Healthy Eating Index 2015; BMI, body mass index; CVD, cardiovascular disease * *P* for trend across the quintile of DAC. HR (95%CI) was estimated by weighted Cox regression analyses Model was adjusted for, age, sex, race, education, family income, dietary energy intake, alcohol consumption per day, smoking status, moderate activity, BMI, diabetes, hypertension, CVD, cancer, hyperlipidaemia, adherence to HEI-2015 score, and dietary supplement use

To further investigate the associations of the meal timing of DAC intake with all-cause mortality, we performed substitution analysis by replacing DAC intake at breakfast with dinner (Fig. 4). The all-cause mortality risk decreased by 7% (aHRs 0.93 [95% CI 0.9–0.97], *P* value = 0.001) when 10% of DAC at breakfast was switched to dinner. We further replaced 10% DAC at breakfast with an equivalent proportion of DAC at dinner by classifying with the main food sources in the substitution model. All-cause mortality did not significantly decrease in models replacing 10% of DAC at breakfast or lunch with 10% of DAC from fruits, grains, dairy products, meats, oils, added sugars, and solid fats at dinner. Conversely, aHRs for all-cause mortality significantly decreased by 5% (aHRs 0.95 [95% CI 0.91–0.99], *P* value = 0.017) in models replacing 10% of DAC at breakfast with 10% of DAC from vegetables at dinner, while that was increased by 7% (aHR 1.07 [95% CI 1.01–1.14], *P* value = 0.022) when substituting 10% of DAC at breakfast with 10% of DAC from alcohol at dinner.

### Regression-based causal mediation analysis

To explore the mechanism underlying the relationship between total, dinner and Δ DACs and all-cause mortality risk, we further obtained 7 biochemical indicators in the NHANES and performed linear regression analysis (Additional file 2: Supplementary Tables 13 and 14). Only CRP among 7 biochemical indicators was significantly associated with total, dinner, and Δ DACs. Therefore, we further examined the associations between serum CRP and meal timing of DAC in quintiles (Additional file 2: Supplementary Fig. 1). Serum CRP was negatively related to total, breakfast, lunch, DAC and Δ DACs (each *P* for trend < 0.05), but the β values for total and dinner DACs were larger than that for breakfast or lunch DACs. Moreover, compared with quintile 1, β values with 95% CIs of serum CRP in quintile 5 were lower for Δ DAC (-0.23 (-0.41, -0.04).

**Fig. 5:**
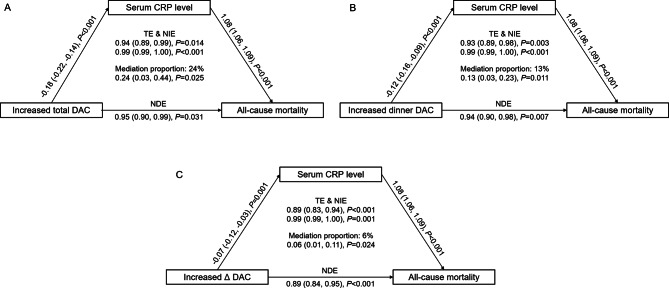
Mediation effects of CRP on associations of total, dinner and Δ DAC with all-cause mortality Abbreviations: HRs, hazard ratio; CIs, confidence intervals; CRP, C-reactive protein; DAC, dietary total antioxidant capacity; TE, total effect; NIE, natural indirect effect; NDE, natural direct effect, HEI-2015, Healthy Eating Index 2015; BMI, body mass index; CVD, cardiovascular disease. HR (95%CI) was estimated by weighted Cox regression analyses. Model was adjusted for, age, sex and race, education, family income, dietary energy intake, alcohol consumption per day, smoking status, physical activity, BMI, diabetes, hypertension, CVD, cancer, hyperlipidaemia, adherence to HEI-2015 score, and dietary supplement use. Models for dinner DAC were further adjusted for breakfast DAC and lunch DAC.

Mediation effects of serum CRP on the association between total, dinner or Δ DACs and risk of all-cause mortality were shown (Fig. 5). Mediation analysis showed that an increase in total DAC decreased the risk of all-cause mortality by 6% (HR [total association], 0.94; 95% CI, 0.89–0.99, *P* = 0.014) and that 24% of the total association (24%; 95% CI, 3-44%) was mediated through serum CRP (HR [indirect association], 0.99; 95% CI, 0.99-1.00, *P* < 0.001). An increase in dinner DAC decreased the risk of all-cause mortality by 7% (HR [total association], 0.93; 95% CI, 0.89–0.98, *P* = 0.003), and 13% of the total association (13%; 95% CI, 3-23%) was mediated through serum CRP (HR [indirect association], 0.99; 95% CI, 0.99-1.00, *P* < 0.001). An increase in Δ DAC decreased the risk of all-cause mortality by 11% (HR [total association], 0.83; 95% CI, 0.89–0.94, *P* < 0.001), and 6% of the total association (6%; 95% CI, 1-11%) was mediated through serum CRP (HR [indirect association], 0.99; 95% CI, 0.99-1.00, *P* = 0.001).

### Sensitivity analysis

In sensitivity analysis, the results did not materially change when replacing the mean values of DAC with median values (Additional file 2: Supplementary Tables 15, 16 and 17). Compared with participants in quintile 1 of total DAC, the all-cause mortality risk in quintile 5 continued to decline (aHRs 0.70 [95% CI 0.61–0.80]), although CVD mortality risk displayed a decreasing tendency (aHRs 0.78 [95% CI 0.59–1.02]). Likewise, the mortality risks for all-cause and CVD were still nonsignificant across breakfast DAC or lunch DAC quintiles. For dinner DAC, all-cause mortality (aHRs 0.77 [95% CI 0.68–0.87]) remained significantly lower in quintile 5 versus quintile 1 and continued to have a nonsignificant association with CVD mortality. For Δ DAC, compared with participants in quintile 1, the all-cause mortality in quintile 5 continued to decline (aHRs 0.82 [95% CI 0.72–0.93]), and the CVD mortality became significantly reduced (aHRs 0.81 [95% CI 0.67–0.99]). The cancer mortality remained nonsignificant in all groups.

The associations of total DAC and dinner DAC with all-cause and CVD mortality were still significant (each *P* < 0.05), and DAC was still not related to cancer mortality when including DAC from midnight snacks. Moreover, midnight DAC consumption was not associated with the risk of death attributed to all causes, CVD and cancer (Additional file 2: Supplementary Table 18).

We also reassessed the associations of DAC with mortality after including snacks, coffee and/or tea (Additional file 2: Supplementary Table 19). When including DAC from snacks, coffee and/or tea, the inverse correlation between total DAC and all-cause and CVD mortality became weakened, but remained significant (each *P* for trend < 0.01), except for a nonsignificant association of tea alone with CVD mortality. For breakfast DAC, when including breakfast DAC from three combination or coffee alone, higher breakfast DAC became associated with a lower risk of all-cause and CVD mortality (each *P* for trend < 0.05). However, when including DAC from breakfast snacks or tea alone, breakfast DAC was still not associated with a lower risk of all-cause, CVD or cancer mortality. For lunch DAC, when including DAC from lunch snacks, coffee and/or tea, lunch DAC was still not associated lower risk of all-cause, CVD and cancer mortality. For dinner DAC, when including DAC from dinner snacks, coffee and/or tea, although the inverse correlation became weakened, higher dinner DAC remained significantly or nominally associated with lower all-cause mortality, but not with CVD or cancer mortality. For Δ DAC, when including Δ DAC from snacks or tea, higher Δ DAC was still associated with lower all-cause mortality (*P* for trend = 0.003). However, when including Δ DAC from coffee, higher Δ DAC was not associated with lower all-cause, CVD or cancer mortality, even some showed a opposite direction.

Finally, the sensitivity analysis was performed using nonimputation data (Additional file 2: Supplementary Table 20). Although total DAC in quintile 5 versus in quintile 1 tended to be related to a reduced all-cause and CVD mortality risk (all-cause: aHRs 0.80 [95% CI 0.62–1.03]; CVD: aHRs 0.82 [95% CI 0.53–1.27]), the higher dinner and Δ DAC in quintile 5 versus in quintile 1 was still associated with a lower risk of all-cause mortality (dinner DAC: aHRs 0.71 [95% CI 0.54–0.93]; Δ DAC: aHRs 0.73 [95% CI 0.57–0.93]). Breakfast and lunch DACs were still not related to mortality.

## Discussion

In this study, we focused on DAC values from three main meals without coffee and calculated the average intakes of DAC, which were consistent with previous studies [12, 43]. We demonstrated that higher total, dinner and Δ DACs without coffee (not breakfast or lunch DACs) were associated with lower all-cause and/or CVD mortality, modified by age, smoking status or income. Furthermore, a higher Δ DAC solely from vegetables was associated with a lower risk of all-cause mortality, while a higher Δ DAC solely from alcohol was associated with greater all-cause and cancer mortality. Mediation analysis further found that serum CRP was a mediator of the association between total, dinner or Δ DACs and the risk of all-cause mortality.

Our findings revealed that total DAC was negatively associated with all-cause mortality in general adult populations, which was supported by a few prior works [12, 14, 15]. Expanding on previous studies, this study further revealed that DAC intake at dinner, but not breakfast or lunch, was negatively related to all-cause mortality. Higher Δ DAC consumption was associated with lower all-cause mortality. Furthermore, this association was independent of traditional risk factors [21, 44], as further demonstrated by sensitivity analysis models. To our knowledge, this study is the first to examine the association of meal timing of DAC with all-cause and specific-cause mortality and to emphasize the importance of DAC distribution for a low risk of mortality. Our findings are supported by a previous study that investigated the potential negative association of meal timing of individual antioxidants on mortality [20]. The optimal intake times of dietary antioxidants vitamin C and vitamin E were in the evening, which was associated with the lowest risks of CVD and all-cause mortalities [20]. Notably, we further found that after including coffee, the inverse associations between total, dinner and Δ DACs and all-cause mortality weakened or was even lost, while an inverse association between breakfast DAC and all-cause mortality was observed. Although previous studies showed that moderate coffee consumption was related to reduced all-cause and cause-specific mortality [45, 46], coffee is probably suitable at breakfast rather than dinner.

Subgroup analysis revealed that age, smoking status or income could modify the association of total, dinner or Δ DACs with all-cause mortality. Antioxidants were associated with reduced risk of age-related disease [47, 48], however, people aged < 65 years were more likely to obtain health benefits from dinner DAC. Likewise, smokers are susceptible to benefit from total and dinner DACs. Consistent with the results, dietary sources of antioxidants significantly reduce the adverse effects of smoking, thus probably being one of the simplest means for smokers to stay healthy [49]. We also found that Δ DAC had a more significant protective effect in high-income groups, which could be attributed to the fact that people with higher incomes tend to lead healthier lifestyles. Cultural-related habits also affect the distribution of meals and health outcomes. For example, traditional rice-based dinners are associated with an increased risk of hyperglycemia [50]. However, the relationship of DAC distribution in different cultural backgrounds with mortality remains largely unknown and merits further study.

Intriguingly, we also found that decreased all-cause mortality risk was found only in the vegetable substitution model but not in the models from fruits, grains, dairy products, meats, oils, added sugars, and solid fats. Conversely, dinner alcohol substitution for breakfast DAC was related to increased all-cause mortality risk. The mortality difference between the consumption distributions of vegetables and other food sources across a day is complex, but caloric differences may explain that in part because vegetables are usually characterized as being low-calorie foods. Indeed, the association of meal timing of certain foods on health has been investigated in abundant studies that consistently demonstrated the risk of high energy intake and alcohol consumption at dinner [21, 30, 51, 52]. Higher intake of energy from dinner rather than breakfast was related to greater CVD and all-cause mortality in diabetes [21]. Moderate alcohol consumption in the evening might predispose patients with type 1 diabetes to hypoglycaemia after breakfast the next morning [30, 51, 52]. Nonetheless, additional research on the health impact of DAC food sources is needed.

Mechanisms underlying the action of dietary antioxidant capacity have been investigated in previous studies, which showed a significant counteractive effect on systemic oxidative stress and inflammation [53, 54]. Mediation analysis found that serum CRP was a mediator of the association between total, dinner or Δ DACs and all-cause mortality risk. The inflammation marker CRP has been shown to be correlated with oxidative stress [55–57]. We speculated that dinner DAC consumption may help reduce oxidative stress and the inflammatory response by synchronizing with circadian rhythms, thereby maximizing health improvement. This assumption may be supported by a series of studies. Meal timing and food composition are routinely shown in the scientific literature to regulate circadian rhythms [58–60]. Moreover, the molecular clock directly exerts its regulatory control over inflammation by fine-tuning various intracellular mechanisms or oxidative stress [61, 62]. Diurnal rhythms were also found in inflammation and oxidative stress factors with a night-time or afternoon peak [63–68]. Obviously, the peak shifts of inflammation are in parallel with shifts in the timing intake of DAC in the evening, probably contributing to the reduction in mortality risk. Combined, our findings suggest that not only DAC values but also meal timing merit adoption in dietary recommendations for the general population.

This study has several strengths. First, this is the first study investigating the association between meal timing of DAC and mortality risk. Furthermore, we adopted the DAC index to reflect the overall antioxidant capacity of the diet considering the synergistic effect of all the antioxidant substances in the different food items. Finally, all available data were derived from the high-quality external dataset NHANES, which is a large prospective cohort with well-designed and validated protocols. Our findings are therefore potentially more generalizable and repeatable.

This study also has several limitations. First, dietary intake was assessed by two nonconsecutive 24-h dietary recalls, which may not fully capture long-term dietary habits. Although the repeatability and effectiveness of the dietary interview were validated, further long-term eating habits should be considered. Second, we were unable to measure the serum DAC level, which would have strengthened our results. Furthermore, although traditional risk factors were adjusted, unmeasured confounding cannot be entirely ruled out. Finally, detailed information on cancer (type, stage or treatment), career and sleep status are lacking, and some information of food sources of DAC is also lacking, which would probably affect the association of DAC with mortality.

## Conclusion

Overall, total DAC from three main meals was negatively associated with all-cause and CVD mortality among adults. More importantly, a higher DAC intake at dinner was associated with lower all-cause mortality independent of traditional risk factors, with special attention given to the coffee and food source of dinner DAC. Furthermore, the inverse association of total, dinner and Δ DAC with all-cause mortality was partially mediated by serum CRP. Our findings emphasize the notion that not only a diet rich in antioxidants but also meal timing is needed to attain survival benefits.

## Electronic supplementary material

Below is the link to the electronic supplementary material.

Additional file 1

Additional file 2

## Data Availability

The data underlying this article will be shared on reasonable request to the corresponding author.

## Abbreviations

HR: hazard ratio
aHRs: adjusted hazard ratios
CI: confidence intervals
NHANES: the US National Health and Nutrition Examination Survey
DAC: dietary total antioxidant capacity
HEI-2015: Healthy Eating Index 2015
BMI: body mass index
CVD: cardiovascular disease
CRP: C-reactive protein
