# Supplementary material for: Meal timing of dietary total antioxidant capacity and its association with all-cause, CVD and cancer mortality: the US national health and nutrition examination survey, 1999–2018
Source: Int J Behav Nutr Phys Act. 2023 Jul 7;20:83. doi: 10.1186/s12966-023-01487-1 (PMC10329360; doi:10.1186/s12966-023-01487-1)
Supplement: Supplementary file 2 — Additional file 2 [file 12966_2023_1487_MOESM2_ESM.pdf]

Additional file 2. Supplementary material and tables

Adjusted covariates selection.

Covariates were selected mainly based on the traditional risk factors and the differential variables from baseline characteristics. We listed the associations of characteristic variables with all-cause of mortality (**Details were shown as follows**). Except for race/ethnicity, physical activity and lunch DAC intake, all other variables were significantly associated with all-cause of mortality. These significant variables were first selected for confounding factors. However, race/ethnicity and physical activity have been widely adjusted for in studies of diet and disease [1,2]. For lunch DAC intake, one study reported that lunch foods intake is related to total daily food intake [3], which indicated the lunch DAC intake may affect breakfast and dinner DAC intake. Therefore, we also adjusted for race/ethnicity, physical activity, and the DAC at lunch. See details as follows:

Characteristic variables	HR (95% CI)	*P* value
Age (years)	1.09 (1.09, 1.10)	<0.0001
Sex	0.88 (0.83, 0.94)	<0.0001
Race/ethnicity	1.00 (0.96, 1.04)	0.963
Education	0.83 (0.79, 0.86)	<0.0001
Income	0.65 (0.62, 0.67)	<0.0001
Body mass index (kg/m2)	1.02 (1.01, 1.03)	<0.0001
Alcohol intake (g/day)	1.00 (1.00, 1.01)	0.001
Smoking status	1.27 (1.21, 1.33)	<0.0001
Physical activity (METs-h/week)	1.00 (0.99, 1.01)	0.551
Dietary energy intake (kcal)	1.00 (1.00, 1.00)	<0.0001
Adherence to HEI-2015 score	0.99 (0.99, 1.00)	<0.0001
Breakfast DAC intake (mmol)	1.06 (1.04, 1.08)	<0.0001
Lunch DAC intake (mmol)	1.00 (0.98, 1.02)	0.915
Dinner DAC intake (mmol)	0.92 (0.89, 0.95)	<0.0001
Dietary supplement use (%)	1.19 (1.11, 1.28)	<0.0001
Hypertension	3.66 (3.38, 3.96)	<0.0001
CVD	4.80 (4.34, 5.30)	<0.0001
Hyperlipidemia	3.39 (3.11, 3.69)	<0.0001
Diabetes	3.77 (3.48, 4.07)	<0.0001
Cancer	3.66 (3.33, 4.03)	<0.0001

Abbreviations: HRs, hazard ratio; CIs, confidence intervals; HEI-2015, Healthy Eating Index 2015; BMI, body mass index; CVD, cardiovascular disease.

Reference

1. Wang P, Song M, Eliassen AH, Wang M, Fung TT, Clinton SK, Rimm EB, Hu FB, Willett WC, Tabung FK et al: Optimal dietary patterns for prevention of chronic disease. Nat Med 2023, 29(3):719-728.
2. Ma L, Hu Y, Alperet DJ, Liu G, Malik V, Manson JE, Rimm EB, Hu FB, Sun Q: Beverage consumption and mortality among adults with type 2 diabetes: prospective cohort study. BMJ 2023, 381:e073406.
3. de Castro JM: When, how much and what foods are eaten are related to total daily food intake. Br J Nutr 2009, 102(8):1228-1237.

	Serum CRP	
Exposure	β value (95% CI)	*P* trend
**Total DAC**		0.001
Q1 (<1.69)	0.00 (Reference)	
Q2 (1.69 to 2.61)	−0.09 (−0.18, 0.00)	
Q3 (2.61 to 3.83)	−0.13 (−0.23, −0.02)	
Q4 (3.83 to 5.82)	−0.20 (−0.30, −0.10)	
Q5 (>5.82)	−0.38 (−0.49, −0.26)	
**Breakfast DAC**		0.011
Q1 (<0.09)	0.00 (Reference)	
Q2 (0.09 to 0.32)	0.10 (0.00, 0.20)	
Q3 (0.32 to 0.65)	0.05 (−0.06, 0.16)	
Q4 (0.65 to 1.77)	0.09 (−0.02, 0.20)	
Q5 (>1.77)	−0.16 (−0.26, −0.06)	
**Lunch DAC**		
Q1 (<0.38)	0.00 (Reference)	0.001
Q2 (0.38 to 0.92)	0.01 (−0.08, 0.10)	
Q3 (0.92 to 1.58)	−0.07 (−0.16, 0.01)	
Q4 (1.58 to 2.73)	−0.10 (−0.20, 0.00)	
Q5 (> 2.73)	−0.14 (−0.25, −0.04)	
**Dinner DAC**		
Q1 (<0.13)	0.00 (Reference)	<0.001
Q2 (0.13 to 0.48)	−0.17 (−0.27, −0.08)	
Q3 (0.48 to 0.98)	−0.18 (−0.27, −0.09)	
Q4 (0.98 to 1.90)	−0.16 (−0.26, −0.06)	
Q5 (>1.90)	−0.32 (−0.43, −0.21)	
**Δ DAC**		
Q1(< −0.96)	0.00 (Reference)	
Q2 (−0.96 to −0.10)	−0.15 (−0.31, 0.01)	0.014
Q3 (−0.10 to 0.39)	−0.21 (−0.45, 0.04)	
Q4 (0.39 to 1.26)	−0.21 (−0.41, −0.01)	
Q5 (> 1.26)	−0.23 (−0.41, −0.04)	

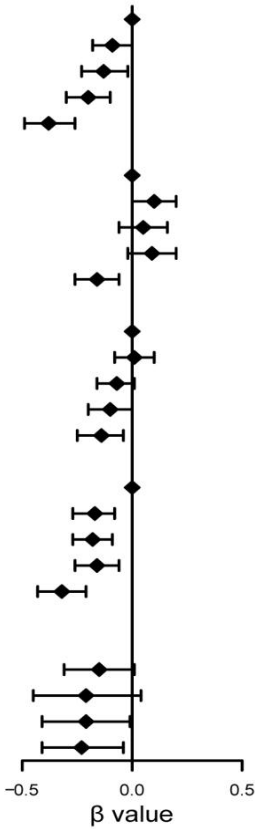

Supplementary Fig. 1 Associations of serum CRP with quintiles of total, breakfast, lunch, dinner, and Δ DAC

Abbreviations: CIs, confidence intervals; CRP, C-reactive protein; DAC, dietary total antioxidant capacity; HEI-2015, Healthy Eating Index 2015; BMI, body mass index; CVD, cardiovascular disease. Model was adjusted for, age, sex, race, education, family income, dietary energy intake, alcohol consumption per day, smoking status, physical activity, BMI, diabetes, hypertension, CVD, cancer, hyperlipidaemia, adherence to HEI-2015 score, and dietary supplement use. Models for breakfast DAC, lunch DAC, and dinner DAC were further adjusted except for the one that defined the group.

Supplementary Table 1 Detailed substitution information of DAC calculation

Food Description	Substitute Information
**Fruits**	
**Citrus, melons, and berries**	
Boysenberries, frozen	Use strawberries instead
Boysenberries, raw	Use strawberries instead
Calamondin, raw	Use orange instead
Cassaba melon, raw	Use honeydew melon instead
Dewberries, raw	Use blackberries instead
Huckleberries, raw	Use the mean value of "Red whortleberries, frozen" and "Red whortleberries, wild" instead
Kumquats, raw	Use orange instead
Loganberries, frozen	Use Raspberries instead
Mulberries, raw	Use Dewberries instead
Tangelos, raw	Use orange instead
Youngberries, raw	Use Dewberries instead
Juneberries, raw	Use the mean value of berries
Loganberries, raw	Use Dewberries instead
**Other fruits**	
Banana flakes, dehydrated	Use "Apples, dried" instead
Carambolas (starfruit), raw	Use the mean value of other fruits
Currants, dried	Use "Figs, dried" instead
Fruit nectar	Use the mean value of less than 100% fruit juice
Fruit, all types, dried, cooked	Use the mean value of all fruit dried
Litchis, dried	Use the mean value of other fruit dried
Litchis, raw	Use the mean value of other fruits

Food Description	Substitute Information
**(continued)**	
Passion fruit, raw	Use "Guava, raw" instead
Peaches, dried	Use the mean value of other fruit dried
Pears, dried	Use the mean value of other fruit dried
Persimmon, raw	Use the mean value of other fruits
Rhubarb, raw	Use the mean value of other fruits
**Grains**	
**Whole grains**	
Amaranth	Use the mean value of whole grain
Millett	Use the mean value of whole grain
Quinoa	Use the mean value of whole grain
Triticale	Use "Wheat" instead
Wild rice	Use "Rice, whole grain" instead
**Refined grains**	
Farina	Use the mean value of refined grain
Masa	Use the mean value of refined grain
Semolina	Use the mean value of refined grain
**Vegetables**	
**Dark Green Vegetables**	
Arugula, raw	Use the mean value of dark green vegetables
Beet greens, cooked	Use the mean value of dark green vegetables
Beet greens, raw	Use the mean value of dark green vegetables
Bitter melon leaves, cooked	Use the mean value of dark green vegetables

Food Description	Substitute Information
**(continued)**	
Cabbage, mustard, cooked	Use "Cabbage, Chinese, cooked" instead
Cabbage, mustard, raw	Use "Cabbage" instead
Chard, cooked	Use the mean value of dark green vegetables
Chicory greens, raw	Use the mean value of dark green vegetables
Chrysanthemum garland, cooked	Use the mean value of dark green vegetables
Cress, cooked, from raw or canned (drained)	Use the mean value of dark green vegetables
Cress, raw	Use "Parsley" instead
Endive, raw	Use the mean value of dark green vegetables
Escarole, cooked	Use the mean value of dark green vegetables
Grape leaves, raw	Use the mean value of dark green vegetables
Lettuce, cooked	Use "Cabbage, Chinese, cooked" instead
Taro leaves, cooked	Use the mean value of dark green vegetables
Turnip greens, cooked, from frozen or canned (drained)	Use the mean value of dark green vegetables
Turnip greens, cooked, from raw	Use the mean value of dark green vegetables
Turnips, cooked, from raw, frozen, or canned (drained)	Use the mean value of dark green vegetables
Watercress, cooked,	Use the mean value of dark green vegetables
Watercress, raw	Use the mean value of dark green vegetables
**Starchy Vegetables**	
Dasheen, cooked	Use the mean value of starchy vegetables
Salsify, cooked	Use the mean value of uncooked starchy vegetables
Tannier, cooked	Use the mean value of uncooked starchy vegetables
Tapioca, pearl, dry	Use the mean value of uncooked starchy vegetables
Taro, cooked	Use the mean value of cooked starchy vegetables
Taro, raw	Use the mean value of uncooked starchy vegetables

Food Description	Substitute Information
**(continued)**	
Water chestnuts, cooked	Use the mean value of cooked starchy vegetables
Water chestnuts, raw	Use the mean value of uncooked starchy vegetables
**Other Red and Orange Vegetables**	
Carrots, dried	Use "carrot,row" instead
Pimiento	Use "Pepper, bell ¯, red" instead
**Other Vegetable**	
Bamboo shoots, cooked	Use the "asparagus, cooked" instead
Bamboo shoots, raw	Use the "asparagus" instead
Bitter melon, cooked	Use the mean value of other vegetables
Breadfruit, raw	Use the mean value of other vegetables
Cactus (Nopales), cooked	Use the mean value of other vegetables
Cactus (Nopales), raw	Use the mean value of other vegetables
Cauliflower, cooked, from canned (drained)	Use the mean value of other vegetables
Celery juice	Use "celery" instead
Chayote (Christophine), cooked	Use the mean value of other vegetables
Chayote (Christophine), raw	Use the mean value of other vegetables
Eggplant, cooked	Use the mean value of other vegetables
Eggplant, pickled	Use the mean value of other vegetables
Escarole, raw	Use the mean value of other vegetables
Fennel bulb, cooked	Use the mean value of other vegetables
Garlic, cooked	Use the "Ginger root, raw" instead
Horseradish tree, leafy tips, cooked	Use the mean value of other vegetables
Jute, potherb, cooked	Use the mean value of other vegetables
Kohlrabi, cooked	Use "Kale, cooked, from raw " instead

Food Description	Substitute Information
**(continued)**	
Kohlrabi, raw	Use "Kale, cooked, from raw " instead
Leeks, cooked	Use the mean value of other vegetables
Mushrooms, dehydrated	NA
Mustard greens, cooked, from frozen or canned (drained)	Use the mean value of other vegetables
Mustard greens, cooked, from raw	Use the mean value of other vegetables
Okra, cooked, from frozen or canned (drained)	Use the mean value of other vegetables
Onions, young green (spring or scallion), raw	Use "Onions" instead
Palm hearts, cooked	Use "Saw Palmetto" instead
Palm hearts, raw	Use "Saw Palmetto" instead
Plantain or green bananas, cooked	Used "banana" instead
Pokeberry shoots, cooked	Use the mean value of other vegetables
Radicchio, raw	Use the mean value of other vegetables
Radish, cooked	Use the mean value of cooked other vegetables
Rutabaga, cooked	Use the mean value of cooked other vegetables
Sauerkraut	Use the mean value of other vegetables
Seaweed, raw	Use the mean value of other vegetables
Snow peas, cooked, from raw or frozen	Use the mean value of cooked other vegetables
Snow peas, raw	Use the mean value of cooked other vegetables
Spinach, cooked, from frozen or canned (drained)	Use the mean value of cooked other vegetables
Squash, spaghetti, cooked	Use the mean value of other vegetables
Squash, summer, cooked, from raw, frozen, or canned (drained)	Use the mean value of other vegetables
String (snap) beans, cooked, from frozen or canned (drained)	Use the mean value of other vegetables
String (snap) beans, cooked, from raw	Use the mean value of other vegetables
String (snap) beans, raw	Use the mean value of other vegetables

Food Description	Substitute Information
**(continued)**	
Taro chips	Use the mean value of uncooked starchy vegetables
Taro leaves, raw	Use the mean value of other vegetables
Winter melon (wax gourd), cooked	Use the mean value of cooked other vegetables
**Beans and Peas**	
Bayo beans, cooked	Use the mean value of cooked beans
Bayo beans, uncooked	Use the mean value of uncooked beans
Black beans, cooked	Use "Black eye beans, haricot blance" instead
Black beans, uncooked	Use the mean value of uncooked beans
Brown beans, cooked	Use the mean value of cooked beans
Brown beans, uncooked	Use the mean value of cooked beans
Calico beans, cooked	Use the mean value of cooked beans
Calico beans, uncooked	Use the mean value of cooked beans
Carob flour or powder	Use the mean value of cooked beans
Chickpeas (garbanzo beans, bengal gram), cooked	Use the mean value of cooked beans
Cowpeas, common (blackeyed, crowder, and southern peas),	Use the mean value of cooked beans
Cowpeas, common (blackeyed, crowder, and southern peas),	Use "Black eye beans, haricot blance" instead
Fava beans (broad beans), cooked	Use "Kidney beans, canned, light red, cooked" instead
Kidney beans, cooked	Use "Kidney beans, canned, light red, cooked" instead
Lentils, cooked	Use "Flageolets beans, green, canned, boiled" instead
Lima beans (mature), cooked	Use the mean value of cooked beans
Lima beans (mature), uncooked	Use the mean value of uncooked beans
Mung beans, cooked	Use the mean value of cooked beans
Split peas, cooked	Use the mean value of cooked beans
White beans, cooked	Use the mean value of cooked beans

Food Description	Substitute Information
**(continued)**	
**Protein Foods**	
**Meat (beef, veal, pork, lamb, gam)**	
Armadillo	Use mean value of meat instead
Bear	Use mean value of meat instead
Beaver	Use mean value of meat instead
Game meat (other)	Use mean value of meat instead
Ham (not cured)	Use pork instead
Opossum	Use mean value of meat instead
Oxtail	Use beef instead
Rabbit	Use mean value of meat instead
Venison	Use "Steak, reindeer" instead
Wild pig	Use pork instead
**Eggs**	
Egg substitute	Use the mean value of "Egg, beaters Egg, scrambled" and "with milk Egg, whites"
**Cured meat**	
Beef luncheon meat	Use the mean value of cured meat instead
Blood sausage	Use the mean value of cured meat instead
**Organ meat**	
Brain	Use the mean value of organ meat instead
Chitterlings	Use the mean value of organ meat instead
Giblets	Use the mean value of organ meat instead
Gizzard	Use the mean value of organ meat instead
Heart	Use the mean value of organ meat instead

Food Description	Substitute Information
**(continued)**	
Kidney	Use the mean value of organ meat instead
Liver	Use the mean value of organ meat instead
Stomach	Use the mean value of organ meat instead
Sweetbreads	Use the mean value of organ meat instead
Thymus	Use the mean value of organ meat instead
Tongue	Use the mean value of organ meat instead
Tripe	Use the mean value of organ meat instead
**Poultry (chicken, turkey, other fowl)**	
Cornish game hen	Use the mean value of chicken instead
Dove	Use the mean value of chicken instead
Duck	Use the mean value of chicken instead
Goose	Use the mean value of chicken instead
Ostrich	Use the mean value of chicken instead
Pheasant	Use the mean value of chicken instead
Quail	Use the mean value of chicken instead
Turkey	Use the mean value of chicken instead
**Beans and peas computed as protein food**	
Miso	Use the mean value of bean protein instead
Natto	Use "soybean" instead
Soybean flour	Use "soybean" instead
Soybean meal	Use "soybean" instead
Soy nuts	Use the mean value of bean protein instead
**Seafood high in n-3 fatty acids**	
Anchovy	Use the mean value of "seafood high in n-3 fatty acids" instead

Food Description	Substitute Information
**(continued)**	
Barracuda	Use the mean value of "seafood high in n-3 fatty acids" instead
Caviar (roe)	Use the mean value of "seafood high in n-3 fatty acids" instead
Cisco	Use the mean value of "seafood high in n-3 fatty acids" instead
Herring	Use the mean value of "seafood high in n-3 fatty acids" instead
Pompano	Use the mean value of "seafood high in n-3 fatty acids" instead
Ray	Use the mean value of "seafood high in n-3 fatty acids" instead
Sardine	Use the mean value of "seafood high in n-3 fatty acids" instead
Sea bass	Use the mean value of "seafood high in n-3 fatty acids" instead
Shad	Use the mean value of "seafood high in n-3 fatty acids" instead
Shark	Use the mean value of "seafood high in n-3 fatty acids" instead
Squid	Use the mean value of "seafood high in n-3 fatty acids" instead
Swordfish	Use the mean value of "seafood high in n-3 fatty acids" instead
Trout	Use the mean value of "seafood high in n-3 fatty acids" instead
Whitefish	Use the mean value of "seafood high in n-3 fatty acids" instead
**Seafood low in n-3 fatty acids**	
Abalone	Use the mean value of "seafood low in n-3 fatty acids" instead
Carp	Use the mean value of "seafood low in n-3 fatty acids" instead
Catfish	Use the mean value of "seafood low in n-3 fatty acids" instead
Crayfish	Use the mean value of "seafood low in n-3 fatty acids" instead
Croaker	Use the mean value of "seafood low in n-3 fatty acids" instead
Eel	Use the mean value of "seafood low in n-3 fatty acids" instead
Flounder	Use the mean value of "seafood low in n-3 fatty acids" instead
Frog legs	Use the mean value of "seafood low in n-3 fatty acids" instead
Haddock	Use the mean value of "seafood low in n-3 fatty acids" instead

Food Description	Substitute Information
**(continued)**	
Halibut	Use the mean value of "seafood low in n-3 fatty acids" instead
Mullet	Use the mean value of "seafood low in n-3 fatty acids" instead
Mussels	Use the mean value of "seafood low in n-3 fatty acids" instead
Ocean perch	Use the mean value of "seafood low in n-3 fatty acids" instead
Octopus	Use the mean value of "seafood low in n-3 fatty acids" instead
Oyster	Use the mean value of "seafood low in n-3 fatty acids" instead
Perch	Use the mean value of "seafood low in n-3 fatty acids" instead
Pike	Use the mean value of "seafood low in n-3 fatty acids" instead
Porgy	Use the mean value of "seafood low in n-3 fatty acids" instead
Scallop	Use the mean value of "seafood low in n-3 fatty acids" instead
Scup	Use the mean value of "seafood low in n-3 fatty acids" instead
Snail	Use the mean value of "seafood low in n-3 fatty acids" instead
Snapper	Use the mean value of "seafood low in n-3 fatty acids" instead
Sole	Use the mean value of "seafood low in n-3 fatty acids" instead
Sturgeon	Use the mean value of "seafood low in n-3 fatty acids" instead
Turtle	Use the mean value of "seafood low in n-3 fatty acids" instead
Whiting	Use the mean value of "seafood low in n-3 fatty acids" instead
**Nuts and seeds**	
Almond butter	Use the mean value of nuts instead
Almond paste	Use the mean value of nuts instead
Cashew butter	Use the mean value of nuts instead
Peanut flour	Use peanuts instead
Pumpkin seeds	Use the mean value of nuts instead
Squash seeds	Use the mean value of nuts instead

Food Description	Substitute Information
**(continued)**	
Sesame butter (tahini)	Use the mean value of nuts instead
Sesame paste	Use the sesame instead
**Dairy**	
Milk, dry, all fat types, not reconstituted	NA
Milk, dry, all fat types, reconstituted	NA
Milk, evaporated, all fat types	Use "Milk, 1%; Milk, 2%" instead
Cheese, blue	Use the mean value of cheese instead
Cheese, brick	Use the mean value of cheese instead
Cheese, brie	Use the mean value of cheese instead
Cheese, camembert	Use the mean value of cheese instead
Cheese, cheddar	Use the mean value of cheese instead
Cheese, colby	Use the mean value of cheese instead
Cheese, cottage variable	Use the mean value of cheese instead
Cheese, cream, fat free	Use the mean value of cheese instead
Cheese, edam	Use the mean value of cheese instead
Cheese, feta	Use the mean value of cheese instead
Cheese, fontina	Use the mean value of cheese instead
Cheese, Mexican, blend, reduced fat	Use "Cheese, Mozzarella, low moisture part skim" instead
Cheese, Mexican, queso anejo	Use the mean value of cheese instead
Cheese, Mexican, queso asadero	Use the mean value of cheese instead
Cheese, Mexican, queso chihuahua	Use the mean value of cheese instead
Cheese, monterey	Use the mean value of cheese instead
Cheese, monterey, low fat	Use the mean value of cheese instead

Food Description	Substitute Information
**(continued)**	
Cheese, muenster	Use the mean value of cheese instead
Cheese, muenster, low fat	Use "Cheese, Mozzarella, low moisture part skim" instead
Cheese, port de salut	Use the mean value of cheese instead
Cheese, provolone	Use the mean value of cheese instead
Cheese, provolone, reduced fat	Use "Cheese, Mozzarella, low moisture part skim" instead
Cheese, queso fresco	Use the mean value of cheese instead
Cheese, ricotta, part skim milk	Use "Cheese, Mozzarella, low moisture part skim" instead
**Oils**	
Cottonseed oil	Use mean value of oils instead
Fish oil	Use mean value of oils instead
Flaxseed oil	Use mean value of oils instead
Peanut oil	Use mean value of oils instead
Sesame oil	Use mean value of oils instead
Spreads	Use mean value of oils instead
Cottonseed oil	Use mean value of oils instead
Fish oil	Use mean value of oils instead
**Solid Fats**	
Cocoa butter	Use mean value of solid fats instead
Cocoa fat	Use mean value of solid fats instead
Coconut cream	Use mean value of solid fats instead
Ghee	Use mean value of solid fats instead
Hydrogenated oils	Use mean value of solid fats instead

Food Description	Substitute Information
**(continued)**	
Lard	Use mean value of solid fats instead
Palm oil	Use mean value of solid fats instead
Tallow	Use mean value of solid fats instead
Shortening (animal and vegetable)	Use mean value of solid fats instead
**Add Sugars**	
Confectioners’sugar	Use the mean value of "syrup (100%), with sugar" instead
Dextrose	Use "Sugar, refined, granulated" instead
Fructose	Use "Sugar, refined, granulated" instead
Fruit juice	Use the mean value of sugars
Fruit syrups	Use the mean value of "syrup (100%), with sugar" instead
Sorghum syrups	Use "Barley malt syrup, organic" instead
Granulated sugar	Sugar, refined, granulated

Supplementary Table 2 The mean and median antioxidant capacity per equivalent serving of the 30 categories foods

Main categories [a]	FPID/FPED categories	Mean Value (mmol/serving) [b]	Median Value (mmol/serving) [c]	Dietary intake servings (Mean (SD)) [d]
**Fruit**	Citrus, melons, and berries	2.696	1.887	0.22 (0.63)
	Other fruits	1.857	1.857	0.29 (0.61)
	Fruit juice	1.182	0.483	0.25 (0.57)
**Vegetables**	Dark green vegetables	1.572	0.590	0.09 (0.31)
	Tomatoes	0.806	0.772	0.26 (0.38)
	Other red and orange vegetables (exclude tomatoes)	0.330	0.147	0.06 (0.19)
	Potatoes (white potatoes)	0.509	0.516	0.29 (0.51)
	Other starchy vegetables (exclude white potatoes)	0.227	0.241	0.07 (0.23)
	Other vegetables	1.182	0.483	0.40 (0.60)
	Beans and peas computed as vegetables	0.561	0.665	0.11 (0.34)
**Grains**	Whole grains	0.173	0.108	0.54 (1.04)
	Refined grains	0.043	0.017	5.04 (3.19)
**Protein Foods**	Meat (beef, veal, pork, lamb, game)	0.008	0.007	1.47 (2.36)
	Cured meat	0.102	0.096	0.77 (1.49)
	Organ meat	0.222	0.237	0.02 (0.31)
	Poultry	0.049	0.037	1.33 (2.32)
	Seafood high in n-3 fatty acids	0.031	0.023	0.10 (0.74)
	Seafood low in n-3 fatty acids	0.031	0.023	0.37 (1.55)
	Eggs	0.027	0.023	0.45 (0.84)

Main categories [a]	FPID/FPED categories	Mean Value (mmol/serving) [b]	Median Value (mmol/serving) [c]	Dietary intake servings (Mean (SD)) [d]
**(continued)**				
	Soybean products	0.056	0.056	0.04 (0.29)
	Nuts and seeds	0.426	0.106	0.22 (0.94)
	Beans and peas computed as protein foods	0.140	0.166	0.45 (1.35)
**Dairy**	Milk (include calcium fortified soy milk)	0.148	0.110	0.73 (0.98)
	Yogurt	0.098	0.098	0.03 (0.14)
	Cheese	0.162	0.028	0.57 (0.84)
**Oils**	Oils	0.668	0.410	16.29 (15.89)
**Solid Fats**	Solid fats	0.339	0.070	31.51 (19.98)
**Added Sugars**	Added sugars	0.008	0.009	12.07 (10.40)
**Alcoholic Drinks**	Alcoholic drinks	1.326	1.960	0.19 (0.96)

[a] Food classification was defined by of USDA's Food Patterns Equivalents Database 2015-2016 (FPED 2015-2016). [b] Mean and [c] median antioxidant capacity value of the food components included in the different food categories defined by the FPED, respectively. [d] The FPED dietary intake servings were calculated by energy adjustment.

Supplementary Table 3 Antioxidant capacity based on food sources at breakfast, lunch, and dinner, respectively

Main Components [a]	FPID/FPED Components	Energy-adjusted antioxidant capacity intake (Mean (SD)) *			
		Total	Breakfast	Lunch	Dinner
**Fruit**	Citrus, melons, and berries	0.46 (1.18)	0.20 (0.82)	0.12 (0.80)	0.11 (0.75)
	Other fruits	0.45 (0.97)	0.19 (0.61)	0.12 (0.52)	0.17 (0.62)
	Fruit juice	0.28 (0.64)	0.15 (0.44)	0.07 (0.32)	0.06 (0.29)
**Vegetables**	Dark green vegetables	0.14 (0.47)	0.01 (0.11)	0.09 (0.37)	0.04 (0.26)
	Tomatoes	0.20 (0.30)	0.02 (0.09)	0.11 (0.23)	0.07 (0.17)
	Other red and orange vegetables (exclude tomatoes)	0.02 (0.06)	0.00 (0.02)	0.01 (0.05)	0.01 (0.04)
	Potatoes (white potatoes)	0.14 (0.26)	0.01 (0.08)	0.08 (0.20)	0.05 (0.14)
	Other starchy vegetables (exclude white potatoes)	0.02 (0.05)	0.00 (0.02)	0.01 (0.04)	0.00 (0.03)
	Other vegetables	0.46 (0.68)	0.03 (0.18)	0.27 (0.53)	0.16 (0.39)
	Beans and peas computed as vegetables	0.06 (0.18)	0.01 (0.07)	0.03 (0.13)	0.02 (0.10)
**Grains**	Whole grains	0.09 (0.18)	0.05 (0.11)	0.02 (0.09)	0.02 (0.08)
	Refined grains	0.21 (0.14)	0.05 (0.07)	0.09 (0.10)	0.07 (0.08)
**Protein Foods**	Meat (beef, veal, pork, lamb, game)	0.01 (0.02)	0.00 (0.00)	0.01 (0.02)	0.00 (0.01)
	Cured meat	0.08 (0.15)	0.02 (0.06)	0.03 (0.09)	0.03 (0.10)
	Organ meat	0.00 (0.07)	0.00 (0.02)	0.00 (0.05)	0.00 (0.03)
	Poultry	0.07 (0.11)	0.00 (0.03)	0.04 (0.09)	0.02 (0.07)
	Seafood high in n-3 fatty acids	0.00 (0.02)	0.00 (0.00)	0.00 (0.02)	0.00 (0.01)
	Seafood low in n-3 fatty acids	0.01 (0.05)	0.00 (0.01)	0.01 (0.04)	0.00 (0.02)
	Eggs	0.01 (0.02)	0.01 (0.02)	0.00 (0.01)	0.00 (0.01)

Main Components [a]	FPID/FPED Components	Energy-adjusted antioxidant capacity intake (Mean (SD)) *			
		Total	Breakfast	Lunch	Dinner
**(continued)**					
	Soybean products	0.00 (0.02)	0.00 (0.01)	0.00 (0.01)	0.00 (0.01)
	Nuts and seeds	0.08 (0.34)	0.02 (0.17)	0.02 (0.20)	0.04 (0.24)
	Beans and peas computed as protein foods	0.06 (0.18)	0.01 (0.07)	0.03 (0.13)	0.02 (0.10)
**Dairy**	Milk (include calcium fortified soy milk)	0.10 (0.14)	0.06 (0.09)	0.02 (0.07)	0.02 (0.06)
	Yogurt	0.00 (0.01)	0.00 (0.01)	0.00 (0.01)	0.00 (0.01)
	Cheese	0.09 (0.13)	0.01 (0.04)	0.05 (0.10)	0.04 (0.08)
**Oils**	Oils	0.11 (0.10)	0.02 (0.04)	0.05 (0.07)	0.04 (0.06)
**Solid Fats**	Solid fats	0.10 (0.07)	0.03 (0.04)	0.05 (0.05)	0.03 (0.04)
**Added Sugars**	Added sugars	0.08 (0.07)	0.02 (0.03)	0.03 (0.05)	0.03 (0.04)
**Alcoholic Drinks**	Alcoholic drinks	0.18 (0.93)	0.01 (0.20)	0.16 (0.98)	0.03 (0.38)

[a] Food classification was defined by of USDA's Food Patterns Equivalents Database 2015-2016 (FPED 2015-2016).

* DAC was obtained by multiplying antioxidant capacity per serving and energy-adjusted dietary intake servings.

Supplementary Table 4 Baseline characteristics of the study population, categorized by quintiles of total DAC

Characteristic	Total DAC					*P* value
	Q1	Q2	Q3	Q4	Q5	
**Patients, n**	11214	11213	11213	11213	11213	
**Age ± years**	40.22±0.28	44.68±0.33	46.82±0.30	48.88±0.31	48.92±0.39	<0.001
**Female**	4662 (41.6)	6053 (54.0)	6047 (53.9)	5984 (53.4)	5187 (46.3)	<0.001
**Race/ethnicity**						<0.001
Mexican American	1477 (13.2)	2002 (17.9)	2022 (18.0)	2030 (18.1)	1783 (15.9)	
Non-Hispanic Black	3173 (28.3)	2531 (22.6)	2172 (19.4)	1880 (16.8)	1655 (14.8)	
Non-Hispanic White	5073 (45.2)	4968 (44.3)	5128 (45.7)	5500 (49.1)	6128 (54.7)	
Other Hispanic	777 (6.9)	910 (8.1)	957 (8.5)	834 (7.4)	671 (6.0)	
Other	714 (6.4)	802 (7.2)	934 (8.3)	969 (8.6)	976 (8.7)	
**Education**						< 0.001
Less than 9th grade	872 (7.8)	1372 (12.2)	1404 (12.5)	1169 (10.4)	967 (8.6)	
9-11th grade	2044 (18.2)	1772 (15.8)	1419 (12.7)	1268 (11.3)	1074 (9.6)	
College graduate or above	1586 (14.2)	1972 (17.6)	2520 (22.5)	3201 (28.6)	4107 (36.7)	
High school graduate/GED or equivalent	3076 (27.4)	2759 (24.6)	2591 (23.1)	2292 (20.5)	1968 (17.6)	
Some college or AA degree	3629 (32.4)	3326 (29.7)	3266 (29.2)	3270 (29.2)	3084 (27.5)	
**Income**						<0.001
$ 0 to $ 19,999	2954 (26.3)	2856 (25.5)	2708 (24.2)	2340 (20.9)	1971 (17.6)	
$20,000 to $44,999	3973 (35.4)	4003 (35.7)	3755 (33.5)	3525 (31.4)	3400 (30.3)	
$45,000 to $74,999	2194 (19.6)	2105 (18.8)	2157 (19.2)	2194 (19.6)	2182 (19.5)	
$75,000 to $99,999	1428 (12.7)	1458 (13.0)	1659 (14.8)	2051 (18.3)	2430 (21.7)	
$100,000 and over	665 (5.9)	791 (7.1)	934 (8.3)	1103 (9.8)	1230 (11.0)	
**BMI (kg/m^2)**	28.52±0.12	29.14±0.12	28.84±0.12	28.32±0.14	27.43±0.12	<0.001
**Alcohol intake (g/day)**	14.06±0.65	6.00±0.36	6.03±0.34	7.59±0.32	15.68±0.53	<0.001

Characteristic	Total DAC					*P* value
	Q1	Q2	Q3	Q4	Q5	
**(continued)**						
**Smoking status**						<0.001
Never smoked	5077 (45.3)	6012 (53.6)	6335 (56.5)	6588 (58.8)	6497 (57.9)	
Past smoker	2142 (19.1)	2653 (23.7)	2844 (25.4)	3085 (27.5)	3350 (29.9)	
Current smoker	3995 (35.6)	2548 (22.7)	2034 (18.1)	1540 (13.7)	1366 (12.2)	
**Physical activity (METs-h/week)**	11.51±0.14	11.74±0.13	11.27±0.13	10.87±0.15	10.28±0.14	<0.001
**Dietary energy intake (kcal)**	2445.54±17.80	1921.01±15.84	1976.51±15.62	2062.22±15.27	2249.79±17.51	<0.001
**Adherence to HEI-2015 score**	42.43±0.21	44.34±0.24	49.87±0.20	54.94±0.24	60.38±0.23	<0.001
**Dietary supplement use (%)**	5753 (51.3)	6120 (54.6)	6526 (58.2)	7088 (63.2)	7559 (67.4)	<0.001
**Serum CRP level (mg/dL)**	5.46±0.23	5.48±0.21	5.34±0.21	5.25±0.24	4.30±0.23	0.001
**Hypertension**	3171 (28.3)	3923 (35.0)	3918 (34.9)	4014 (35.8)	3559 (31.7)	<0.001
**Diabetes**	1444 (12.9)	1880 (16.8)	2119 (18.9)	1986 (17.7)	1541 (13.7)	<0.001
**Cancer**	706 (6.3)	923 (8.2)	1160 (10.3)	1113 (9.9)	1175 (10.5)	<0.001
**Hyperlipidaemia**	1302 (11.6)	1809 (16.1)	2105 (18.8)	2278 (20.3)	1989 (17.7)	<0.001
**CVD**	2830 (25.2)	3477 (31.0)	3673 (32.8)	3664 (32.7)	3406 (30.4)	<0.001

Abbreviations: DAC, dietary total antioxidant capacity; HEI-2015, Healthy Eating Index 2015; BMI, body mass index; CVD, cardiovascular disease; CRP, C-reactive protein.

Continuous variables were adjusted for survey weights of NHANES. Categorical variables were unweighted. One-way ANOVA for continuous variables and chi-square test of independence test for categorical variables were performed.

Supplementary Table 5 Baseline characteristics of the study population, categorized by quintiles of DAC at dinner

Characteristic	DAC at dinner					*P* value
	Q1	Q2	Q3	Q4	Q5	
**Patients, n**	11214	11213	11213	11213	11213	
**Age (years)**	44.33±0.34	47.72±0.28	45.65±0.28	45.64±0.33	46.53±0.37	<0.001
**Female**	3882 (34.6)	6324 (56.4)	6099 (54.4)	6022 (53.7)	5606 (50.0)	<0.001
**Race/ethnicity**						<0.001
Mexican American	1807 (16.1)	1630 (14.5)	1659 (14.8)	1994 (17.8)	2224 (19.8)	
Non-Hispanic Black	2772 (24.7)	2819 (25.1)	2119 (18.9)	1946 (17.4)	1755 (15.7)	
Non-Hispanic White	4942 (44.1)	5062 (45.1)	5881 (52.4)	5418 (48.3)	5494 (49.0)	
Other Hispanic	1080 (9.6)	972 (8.7)	691 (6.2)	736 (6.6)	670 (6.0)	
Other	613 (5.5)	730 (6.5)	863 (7.7)	1119 (10.0)	1070 (9.5)	
**Education**						<0.001
Less than 9th grade	1090 (9.7)	1416 (12.6)	1003 (8.9)	1100 (9.8)	1175 (10.5)	
9-11th grade	1839 (16.4)	1844 (16.5)	1477 (13.2)	1248 (11.1)	1169 (10.4)	
College graduate or above	1968 (17.6)	2026 (18.1)	2599 (23.2)	3124 (27.9)	3669 (32.8)	
High school graduate/GED or equivalent	2847 (25.4)	2734 (24.4)	2702 (24.1)	2361 (21.1)	2042 (18.2)	
Some college or AA degree	3456 (30.9)	3178 (28.4)	3426 (30.6)	3373 (30.1)	3142 (28.1)	
**Income**						<0.001
$ 0 to $ 19,999	2762 (24.6)	3081 (27.5)	2450 (21.8)	2362 (21.1)	2174 (19.4)	
$20,000 to $44,999	3875 (34.6)	3951 (35.2)	3775 (33.7)	3554 (31.7)	3501 (31.2)	
$45,000 to $74,999	2242 (20.0)	2019 (18.0)	2180 (19.4)	2201 (19.6)	2190 (19.5)	
$75,000 to $99,999	1570 (14.0)	1463 (13.0)	1856 (16.6)	1932 (17.2)	2205 (19.7)	
$100,000 and Over	765 (6.8)	699 (6.2)	952 (8.5)	1164 (10.4)	1143 (10.2)	
**BMI (kg/m^2)**	28.78±0.13	28.66±0.12	28.48±0.13	28.41±0.12	27.87±0.12	<0.001
**Alcohol intake (g/day)**	15.85±0.68	8.11±0.41	8.81±0.48	7.75±0.32	9.99±0.44	<0.001

Characteristic	DAC at dinner					*P* value
	Q1	Q2	Q3	Q4	Q5	
**(continued)**						
**Smoking status**						<0.001
Never smoked	5216 (46.5)	5783 (51.6)	6156 (54.9)	6701 (59.8)	6653 (59.3)	
Past smoker	2728 (24.3)	2798 (25.0)	2797 (24.9)	2700 (24.1)	3051 (27.2)	
Current smoker	3270 (29.2)	2632 (23.5)	2260 (20.2)	1812 (16.2)	1509 (13.5)	
**Physical activity (METs-h/week)**	11.54±0.11	11.66±0.13	11.21±0.13	10.87±0.14	10.42±0.13	<0.001
**Dietary energy intake (kcal)**	2588.78±13.17	1791.75±17.81	2019.66±17.97	2091.14±16.46	2196.42±17.45	<0.001
**Adherence to HEI-2015 score**	47.93±0.27	47.78±0.31	48.31±0.27	51.13±0.26	57.87±0.29	<0.001
**Dietary supplement use (%)**	6211 (55.4)	6455 (57.6)	6597 (58.8)	6650 (59.3)	7133 (63.6)	<0.001
**Serum CRP level (mg/dL)**	5.40±0.21	5.24±0.22	5.17±0.21	5.24±0.18	4.75±0.19	0.12
**Hypertension**	3435 (30.6)	4256 (38.0)	3749 (33.4)	3585 (32.0)	3560 (31.7)	<0.001
**Diabetes**	1573 (14.0)	2094 (18.7)	1850 (16.5)	1768 (15.8)	1685 (15.0)	<0.001
**Cancer**	871 (7.8)	1099 (9.8)	1108 (9.9)	993 (8.9)	1006 (9.0)	<0.001
**Hyperlipidaemia**	1657 (14.8)	2081 (18.6)	1913 (17.1)	1826 (16.3)	2006 (17.9)	<0.001
**CVD**	3134 (27.9)	3946 (35.2)	3580 (31.9)	3186 (28.4)	3204 (28.6)	<0.001

Abbreviations: DAC, dietary total antioxidant capacity; HEI-2015, Healthy Eating Index 2015; BMI, body mass index; CVD, cardiovascular disease; CRP, C-reactive protein.

Continuous variables were adjusted for survey weights of NHANES. Categorical variables were unweighted. One-way ANOVA for continuous variables and chi-square test of independence test for categorical variables were performed.

Supplementary Table 6 Baseline characteristics of the study population, categorized by the Δ DAC

Characteristic	Δ DAC [a]					*P* value
	Q1	Q2	Q3	Q4	Q5	
**Patients, n**	11,214	11,213	11,213	11,213	11,213	
**Age (years)**	48.88±0.39	47.80±0.35	45.55±0.29	44.01±0.30	44.24±0.33	< 0.001
**Female**	4053 (36.6)	6103 (55.3)	6141 (55.5)	6195 (56.0)	5212 (47.1)	<0.001
**Race/ethnicity**						<0.001
Mexican American	2169 (19.3)	1761 (15.7)	1421 (12.7)	1795 (16.0)	2168 (19.3)	
Non-Hispanic Black	2006 (17.9)	2591 (23.1)	2755 (24.6)	2169 (19.3)	1890 (16.9)	
Non-Hispanic White	5130 (45.7)	5290 (47.2)	5476 (48.8)	5594 (49.9)	5307 (47.3)	
Other Hispanic	1259 (11.2)	857 (7.6)	662 (5.9)	674 (6.0)	697 (6.2)	
Other	650 (5.8)	714 (6.4)	899 (8.0)	981 (8.7)	1151 (10.3)	
**Education**						< 0.001
Less than 9th grade	1360 (12.1)	1326 (11.8)	961 (8.6)	1003 (8.9)	1134 (10.1)	
9-11th grade	1469 (13.1)	1734 (15.5)	1763 (15.7)	1361 (12.1)	1250 (11.2)	
College graduate or above	2918 (26.0)	2271 (20.3)	2234 (19.9)	2654 (23.7)	3309 (29.5)	
High school graduate/GED or equivalent	2316 (20.7)	2653 (23.7)	2818 (25.2)	2690 (24.0)	2209 (19.7)	
Some college or AA degree	3139 (28.0)	3214 (28.7)	3425 (30.6)	3500 (31.2)	3297 (29.4)	
**Income**						< 0.001
$ 0 to $ 19,999	2505 (22.3)	2882 (25.7)	2850 (25.4)	2425 (21.6)	2167 (19.3)	
$20,000 to $44,999	3797 (33.9)	3776 (33.7)	3765 (33.6)	3660 (32.6)	3658 (32.6)	
$45,000 to $74,999	2187 (19.5)	2123 (18.9)	2149 (19.2)	2193 (19.6)	2180 (19.4)	
$75,000 to $99,999	1876 (16.7)	1628 (14.5)	1600 (14.3)	1870 (16.7)	2052 (18.3)	
$100,000 and Over	849 (7.6)	804 (7.2)	849 (7.6)	1065 (9.5)	1156 (10.3)	
**BMI (kg/m^2)**	27.93±0.13	28.64±0.12	28.83±0.16	28.50±0.13	28.22±0.12	0.110
**Alcohol intake (g/day)**	9.53±0.48	10.44±0.51	10.05±0.52	8.96±0.41	11.08±0.45	<0.001

Characteristic	Δ DAC [a]					*P* value
	Q1	Q2	Q3	Q4	Q5	
**(continued)**						
**Smoking status**						<0.001
Never smoked	6548 (58.4)	5797 (51.7)	5549 (49.5)	6134 (54.7)	6481 (57.8)	
Past smoker	3105 (27.7)	2938 (26.2)	2617 (23.3)	2583 (23.0)	2831 (25.2)	
Current smoker	1561 (13.9)	2478 (22.1)	3047 (27.2)	2496 (22.3)	1901 (17.0)	
**Physical activity (METs-h/week)**	10.89±0.13	11.50±0.15	11.64±0.14	10.97±0.14	10.65±0.13	<0.001
**Dietary energy intake (kcal)**	2240.88±18.83	2145.52±16.39	1912.36±13.61	2113.68±17.24	2268.24±18.28	<0.001
**Adherence to HEI-2015 score**	56.73±0.21	49.15±0.30	46.78±0.28	47.66±0.30	53.77±0.27	<0.001
**Dietary supplement use (%)**	7119 (63.5)	6530 (58.2)	6262 (55.8)	6447 (57.5)	6688 (59.6)	<0.001
**Serum CRP level (mg/dL)**	4.66±0.21	5.63±0.23	5.51±0.24	5.06±0.19	4.92±0.21	0.002
**Hypertension**	3774 (33.7)	4081 (36.4)	3848 (34.3)	3500 (31.2)	3382 (30.2)	<0.001
**Diabetes**	1754 (15.6)	2048 (18.3)	1860 (16.6)	1694 (15.1)	1614 (14.4)	<0.001
**Cancer**	1192 (10.6)	1117 (10.0)	1055 (9.4)	875 (7.8)	838 (7.5)	<0.001
**Hyperlipidaemia**	1995 (17.8)	2129 (19.0)	1866 (16.6)	1681 (15.0)	1812 (16.2)	<0.001
**CVD**	3606 (32.2)	3861 (34.4)	3584 (32.0)	3087 (27.5)	2912 (26.0)	<0.001

Abbreviations: DAC, dietary total antioxidant capacity; HEI-2015, Healthy Eating Index 2015; BMI, body mass index; CVD, cardiovascular disease; CRP, C-reactive protein.

Continuous variables were adjusted for survey weights of NHANES. Categorical variables were unweighted. One-way ANOVA for continuous variables and chi-square test of independence test for categorical variables were performed.

[a] Δ equals dinner DAC minus breakfast DAC.

Supplementary Table 7 Associations of all-cause mortality with quintiles of total, breakfast, lunch, dinner, and Δ DAC

Exposure	Case/N	Model 1		Model 2		Model 3	
		HR (95% CI)	*P* value	HR (95% CI)	*P* value	HR (95% CI)	*P* value
**Total DAC**							
Continuous total DAC	8566/56066	0.93 (0.91, 0.94)	<0.001	0.95 (0.94, 0.97)	<0.001	0.96 (0.95, 0.98)	<0.001
Quintile 1	1278/11214	1.00 (Reference)		1.00 (Reference)		1.00 (Reference)	
Quintile 2	1613/11213	0.75 (0.65, 0.87)		0.81 (0.69, 0.94)		0.82 (0.70, 0.95)	
Quintile 3	1896/11213	0.73 (0.64, 0.84)		0.82 (0.72, 0.95)		0.83 (0.71, 0.95)	
Quintile 4	1896/11213	0.59 (0.51, 0.68)		0.70 (0.61, 0.81)		0.74 (0.63, 0.85)	
Quintile 5	1883/11213	0.48 (0.43, 0.54)	<0.001*	0.62 (0.54, 0.70)	<0.001*	0.66 (0.57, 0.76)	<0.001*
**Breakfast DAC**							
Continuous breakfast DAC	8566/56066	0.93 (0.91, 0.95)	<0.001	0.98 (0.96, 1.00)	0.049	1.00 (0.98, 1.02)	0.777
Quintile 1	929/11214	1.00 (Reference)		1.00 (Reference)		1.00 (Reference)	
Quintile 2	1470/11213	0.93 (0.80, 1.08)		0.91 (0.79, 1.06)		0.92 (0.79, 1.07)	
Quintile 3	1974/11213	0.86 (0.76, 0.98)		0.87 (0.75, 1.00)		0.87 (0.75, 1.01)	
Quintile 4	1914/11213	0.81 (0.70, 0.93)		0.91 (0.78, 1.06)		0.93 (0.79, 1.08)	
Quintile 5	2279/11213	0.74 (0.64, 0.85)	<0.001*	0.89 (0.77, 1.03)	0.267*	0.96 (0.82, 1.12)	0.976*
**Lunch DAC**							
Continuous lunch DAC	8566/56066	0.93 (0.92, 0.95)	<0.001	0.96 (0.94, 0.98)	<0.001	0.96 (0.94, 0.99)	0.002
Quintile 1	1227/11214	1.00 (Reference)		1.00 (Reference)		1.00 (Reference)	
Quintile 2	1788/11213	1.14 (1.01, 1.28)		1.05 (0.93, 1.19)		1.00 (0.89, 1.13)	
Quintile 3	1828/11213	1.00 (0.89, 1.14)		1.00 (0.88, 1.14)		0.97 (0.86, 1.10)	
Quintile 4	1967/11213	0.95 (0.84, 1.08)		1.00 (0.88, 1.14)		0.97 (0.85, 1.10)	
Quintile 5	1756/11213	0.77 (0.69, 0.86)	<0.001*	0.88 (0.78, 1.00)	0.023*	0.88 (0.77, 1.01)	0.054*

Exposure	Case/N	Model 1		Model 2		Model 3	
		HR (95% CI)	*P* value	HR (95% CI)	*P* value	HR (95% CI)	*P* value
**(continued)**							
**Dinner DAC**							
Continuous dinner DAC	8566/56066	0.90 (0.88, 0.93)	<0.001	0.95 (0.92, 0.98)	<0.001	0.95 (0.92, 0.98)	0.001
Quintile 1	1537/11214	1.00 (Reference)		1.00 (Reference)		1.00 (Reference)	
Quintile 2	2103/11213	0.97 (0.86, 1.10)		0.95 (0.84, 1.08)		0.91 (0.81, 1.03)	
Quintile 3	1795/11213	0.88 (0.78, 0.99)		0.93 (0.83, 1.04)		0.87 (0.78, 0.97)	
Quintile 4	1568/11213	0.79 (0.69, 0.91)		0.88 (0.77, 1.01)		0.83 (0.72, 0.95)	
Quintile 5	1563/11213	0.67 (0.58, 0.77)	<0.001*	0.79 (0.69, 0.90)	<0.001*	0.76 (0.67, 0.87)	<0.001*
**Δ DAC**							
Continuous Δ DAC	8566/56066	0.99 (0.98, 1.01)	0.393	0.99 (0.97, 1.00)	0.105	0.98 (0.97, 1.00)	0.036
Quintile 1	2144/11214	1.00 (Reference)		1.00 (Reference)		1.00 (Reference)	
Quintile 2	2005/11213	1.16 (1.04, 1.30)		1.04 (0.93, 1.15)		0.99 (0.90, 1.10)	
Quintile 3	1779/11213	1.28 (1.13, 1.44)		1.10 (0.98, 1.23)		1.03 (0.92, 1.17)	
Quintile 4	1364/11213	1.04 (0.93, 1.16)		0.96 (0.87, 1.07)		0.91 (0.82, 1.01)	
Quintile 5	1274/11213	0.87 (0.76, 0.99)	0.022*	0.85 (0.75, 0.97)	0.008*	0.84 (0.74, 0.96)	0.003*

Abbreviations: HRs, hazard ratios; CIs, confidence intervals; DAC, dietary total antioxidant capacity; HEI-2015, Healthy Eating Index 2015; BMI, body mass index; CVD, cardiovascular disease.

* *P* for trend across the quintile of DAC. HR (95%CI) was estimated by weighted Cox regression analyses. Δ equals dinner DAC minus breakfast DAC.

Model 1 adjusted for, age, sex, and race.

Model 2 further adjusted for education, family income, dietary energy intake, alcohol consumption per day, smoking status, physical activity, and BMI.

Model 3 further adjusted for diabetes, hypertension, CVD, cancer, hyperlipidaemia, adherence to HEI-2015 score, and dietary supplement use.

Models for breakfast DAC, lunch DAC, and dinner DAC were further adjusted except for the one that defined the group.

Supplementary Table 8 Associations of CVD mortality with quintiles of total, breakfast, lunch, dinner, and Δ DAC

Exposure	Case/N	Model 1		Model 2		Model 3	
		HR (95% CI)	*P* value	HR (95% CI)	*P* value	HR (95% CI)	*P* value
**Total DAC**							
Continuous total DAC	2196/56066	0.92 (0.90, 0.94)	<0.001	0.96 (0.93, 0.98)	0.001	0.98 (0.95, 1.01)	0.144
Quintile 1	293/11214	1.00 (Reference)		1.00 (Reference)		1.00 (Reference)	
Quintile 2	417/11213	0.84 (0.63, 1.11)		0.86 (0.65, 1.15)		0.86 (0.65, 1.15)	
Quintile 3	495/11213	0.80 (0.60, 1.07)		0.87 (0.63, 1.20)		0.90 (0.66, 1.23)	
Quintile 4	495/11213	0.68 (0.52, 0.88)		0.81 (0.61, 1.06)		0.90 (0.68, 1.18)	
Quintile 5	496/11213	0.47 (0.38, 0.59)	<0.001*	0.62 (0.49, 0.79)	<0.001*	0.73 (0.57, 0.94)	0.059*
**Breakfast DAC**							
Continuous breakfast DAC	2196/56066	0.90 (0.87, 0.94)	<0.001	0.96 (0.92, 1.00)	0.038	0.99 (0.95, 1.03)	0.593
Quintile 1	203/11214	1.00 (Reference)		1.00 (Reference)		1.00 (Reference)	
Quintile 2	365/11213	1.02 (0.75, 1.40)		0.94 (0.66, 1.34)		0.93 (0.66, 1.32)	
Quintile 3	533/11213	0.90 (0.70, 1.15)		0.83 (0.63, 1.08)		0.82 (0.63, 1.07)	
Quintile 4	495/11213	0.84 (0.64, 1.10)		0.88 (0.66, 1.17)		0.91 (0.68, 1.22)	
Quintile 5	600/11213	0.71 (0.56, 0.89)	<0.001*	0.82 (0.64, 1.05)	0.079*	0.93 (0.72, 1.20)	0.740*
**Lunch DAC**							
Continuous lunch DAC	2196/56066	0.95 (0.91, 0.99)	0.008	0.99 (0.96, 1.03)	0.71	1.00 (0.96, 1.04)	0.972
Quintile 1	270/11214	1.00 (Reference)		1.00 (Reference)		1.00 (Reference)	
Quintile 2	458/11213	1.31 (0.99, 1.74)		1.15 (0.87, 1.51)		1.08 (0.82, 1.41)	
Quintile 3	523/11213	1.35 (1.03, 1.78)		1.32 (1.01, 1.73)		1.28 (0.98, 1.66)	
Quintile 4	505/11213	1.13 (0.87, 1.47)		1.17 (0.90, 1.53)		1.13 (0.88, 1.45)	
Quintile 5	440/11213	0.88 (0.67, 1.15)	0.016*	1.05 (0.81, 1.38)	0.962*	1.07 (0.82, 1.40)	0.660*

Exposure	Case/N	Model 1		Model 2		Model 3	
		HR (95% CI)	*P* value	HR (95% CI)	*P* value	HR (95% CI)	*P* value
**(continued)**							
**Dinner DAC**							
Continuous dinner DAC	2196/56066	0.88 (0.84, 0.93)	<0.001	0.93 (0.88, 0.98)	0.003	0.95 (0.90, 1.00)	0.039
Quintile 1	363/11214	1.00 (Reference)		1.00 (Reference)		1.00 (Reference)	
Quintile 2	550/11213	0.95 (0.77, 1.18)		0.87 (0.70, 1.09)		0.83 (0.66, 1.03)	
Quintile 3	475/11213	0.92 (0.73, 1.16)		0.92 (0.72, 1.17)		0.87 (0.68, 1.11)	
Quintile 4	397/11213	0.84 (0.66, 1.05)		0.88 (0.69, 1.12)		0.86 (0.68, 1.09)	
Quintile 5	411/11213	0.66 (0.52, 0.84)	<0.001*	0.76 (0.60, 0.96)	0.029*	0.78 (0.61, 1.00)	0.118*
**Δ DAC**							
Continuous Δ DAC	2196/56066	1.00 (0.98, 1.02)	0.899	0.99 (0.97, 1.02)	0.548	0.98 (0.96, 1.01)	0.221
Quintile 1	554/11214	1.00 (Reference)		1.00 (Reference)		1.00 (Reference)	
Quintile 2	527/11213	1.32 (1.09, 1.59)		1.15 (0.94, 1.39)		1.06 (0.88, 1.28)	
Quintile 3	444/11213	1.34 (1.11, 1.62)		1.12 (0.92, 1.37)		1.00 (0.82, 1.23)	
Quintile 4	325/11213	1.02 (0.82, 1.26)		0.91 (0.74, 1.13)		0.84 (0.68, 1.04)	
Quintile 5	346/11213	1.02 (0.84, 1.23)	0.558*	0.99 (0.82, 1.20)	0.342*	0.96 (0.80, 1.16)	0.177*

Abbreviations: HRs, hazard ratio; CIs, confidence intervals; DAC, dietary total antioxidant capacity; HEI-2015, Healthy Eating Index 2015; BMI, body mass index; CVD, cardiovascular disease.

* *P* for trend across the quintile of DAC. HR (95%CI) was estimated by weighted Cox regression analyses. Δ equals dinner DAC minus breakfast DAC.

Model 1 adjusted for, age, sex, and race.

Model 2 further adjusted for education, family income, dietary energy intake, alcohol consumption per day, smoking status, physical activity, and BMI.

Model 3 further adjusted for diabetes, hypertension, CVD, cancer, hyperlipidaemia, adherence to HEI-2015 score, and dietary supplement use.

Models for breakfast DAC, lunch DAC, and dinner DAC were further adjusted except for the one that defined the group.

Supplementary Table 9 Associations of cancer mortality with quintiles of total, breakfast, lunch, dinner, and Δ DAC

Exposure	Case/N	Model 1		Model 2		Model 3	
		HR (95% CI)	*P* value	HR (95% CI)	*P* value	HR (95% CI)	*P* value
**Total DAC**							
Continuous total DAC	1984/56066	0.95 (0.92, 0.98)	0.001	0.97 (0.94, 1.00)	0.082	0.98 (0.94, 1.02)	0.256
Quintile 1	315/11214	1.00 (Reference)		1.00 (Reference)		1.00 (Reference)	
Quintile 2	401/11213	0.97 (0.73, 1.29)		1.00 (0.75, 1.34)		1.04 (0.77, 1.39)	
Quintile 3	425/11213	0.86 (0.66, 1.12)		0.93 (0.71, 1.22)		0.94 (0.71, 1.25)	
Quintile 4	435/11213	0.69 (0.55, 0.85)		0.78 (0.62, 0.98)		0.81 (0.62, 1.05)	
Quintile 5	408/11213	0.61 (0.48, 0.78)	<0.001*	0.72 (0.56, 0.94)	0.001*	0.76 (0.56, 1.03)	0.019*
**Breakfast DAC**							
Continuous breakfast DAC	1984/56066	0.93 (0.88, 0.98)	0.012	0.97 (0.91, 1.02)	0.225	0.98 (0.92, 1.04)	0.453
Quintile 1	250/11214	1.00 (Reference)		1.00 (Reference)		1.00 (Reference)	
Quintile 2	375/11213	1.11 (0.84, 1.48)		1.02 (0.76, 1.37)		1.02 (0.77, 1.37)	
Quintile 3	430/11213	0.84 (0.65, 1.09)		0.80 (0.60, 1.06)		0.81 (0.61, 1.07)	
Quintile 4	444/11213	0.92 (0.72, 1.17)		0.96 (0.75, 1.24)		1.00 (0.77, 1.29)	
Quintile 5	485/11213	0.76 (0.57, 1.02)	0.005*	0.85 (0.63, 1.15)	0.217*	0.90 (0.65, 1.24)	0.501*
**Lunch DAC**							
Continuous lunch DAC	1984/56066	0.96 (0.92, 1.01)	0.096	0.98 (0.94, 1.03)	0.484	0.99 (0.95, 1.04)	0.667
Quintile 1	326/11214	1.00 (Reference)		1.00 (Reference)		1.00 (Reference)	
Quintile 2	389/11213	1.19 (0.94, 1.51)		1.06 (0.84, 1.35)		1.03 (0.81, 1.31)	
Quintile 3	412/11213	1.04 (0.82, 1.33)		0.96 (0.75, 1.23)		0.92 (0.72, 1.19)	
Quintile 4	451/11213	1.07 (0.81, 1.40)		1.03 (0.78, 1.37)		1.03 (0.78, 1.35)	
Quintile 5	406/11213	0.98 (0.75, 1.27)	0.449*	1.03 (0.78, 1.36)	0.909*	1.07 (0.79, 1.43)	0.671*

Exposure	Case/N	Model 1		Model 2		Model 3	
		HR (95% CI)	*P* value	HR (95% CI)	*P* value	HR (95% CI)	*P* value
**(continued)**							
**Dinner DAC**							
Continuous dinner DAC	1984/56066	0.96 (0.90, 1.03)	0.233	0.99 (0.93, 1.06)	0.854	1.00 (0.93, 1.07)	0.996
Quintile 1	384/11214	1.00 (Reference)		1.00 (Reference)		1.00 (Reference)	
Quintile 2	484/11213	1.12 (0.89, 1.40)		1.03 (0.82, 1.31)		1.00 (0.79, 1.27)	
Quintile 3	384/11213	0.84 (0.67, 1.06)		0.84 (0.66, 1.07)		0.80 (0.63, 1.02)	
Quintile 4	354/11213	0.87 (0.65, 1.15)		0.91 (0.69, 1.19)		0.86 (0.67, 1.12)	
Quintile 5	378/11213	0.80 (0.61, 1.06)	0.023*	0.88 (0.67, 1.17)	0.225*	0.87 (0.66, 1.15)	0.171*
**Δ DAC value**							
Continuous Δ DAC value	1984/56066	1.02 (0.98, 1.06)	0.385	1.02 (0.97, 1.06)	0.519	1.01 (0.97, 1.06)	0.561
Quintile 1	476/11214	1.00 (Reference)		1.00 (Reference)		1.00 (Reference)	
Quintile 2	442/11213	1.03 (0.83, 1.27)		0.93 (0.75, 1.14)		0.90 (0.73, 1.13)	
Quintile 3	403/11213	1.21 (0.97, 1.52)		1.03 (0.82, 1.28)		0.98 (0.75, 1.27)	
Quintile 4	337/11213	1.08 (0.87, 1.34)		0.99 (0.80, 1.24)		0.96 (0.76, 1.22)	
Quintile 5	326/11213	0.92 (0.70, 1.21)	0.794*	0.90 (0.69, 1.19)	0.673*	0.90 (0.68, 1.20)	0.632*

Abbreviations: HRs, hazard ratio; CIs, confidence intervals; DAC, dietary total antioxidant capacity; HEI-2015, Healthy Eating Index 2015; BMI, body mass index; CVD, cardiovascular disease.

* *P* for trend across the quintile of DAC. HR (95%CI) was estimated by weighted Cox regression analyses. Δ equals dinner DAC minus breakfast DAC.

Model 1 adjusted for, age, sex, and race.

Model 2 further adjusted for education, family income, dietary energy intake, alcohol consumption per day, smoking status, physical activity, and BMI.

Model 3 further adjusted for diabetes, hypertension, CVD, cancer, hyperlipidaemia, adherence to HEI-2015 score, and dietary supplement use.

Models for breakfast DAC, lunch DAC, and dinner DAC were further adjusted except for the one that defined the group.

Supplementary Table 10 Association of total DAC and all-cause mortality by ten characteristics

Characteristics	Case/N	Total DAC					*P* for interaction
		Quintile 1	Quintile 2	Quintile 3	Quintile 4	Quintile 5	
**Age (years)**							0.133
age<65	2671/43027	1.00 (Reference)	0.99 (0.78, 1.26)	1.10 (0.86, 1.40)	1.01 (0.78, 1.29)	1.02 (0.78, 1.33)	
age≥65	5895/13039	1.00 (Reference)	0.85 (0.73, 0.99)	0.94 (0.80, 1.10)	0.90 (0.75, 1.08)	0.74 (0.61, 0.89)	
**Sex**							0.152
Men	4832/28133	1.00 (Reference)	0.88 (0.73, 1.06)	0.91 (0.74, 1.12)	0.80 (0.67, 0.96)	0.64 (0.52, 0.80)	
Women	3734/27933	1.00 (Reference)	0.80 (0.64, 0.98)	0.82 (0.67, 1.01)	0.75 (0.60, 0.93)	0.77 (0.59, 0.99)	
**Race/ethnicity**							0.935
Mexican American	1074/9314	1.00 (Reference)	1.00 (0.67, 1.49)	1.15 (0.82, 1.60)	0.94 (0.59, 1.48)	1.31 (0.70, 2.44)	
Non-Hispanic Black	1630/11411	1.00 (Reference)	0.91 (0.72, 1.15)	1.07 (0.81, 1.41)	0.96 (0.73, 1.27)	0.82 (0.53, 1.27)	
Non-Hispanic White	5261/26797	1.00 (Reference)	0.82 (0.68, 0.98)	0.82 (0.68, 0.98)	0.72 (0.60, 0.85)	0.61 (0.51, 0.73)	
Other Hispanic	334/4149	1.00 (Reference)	1.00 (0.53, 1.87)	0.98 (0.50, 1.92)	1.43 (0.54, 3.82)	1.38 (0.53, 3.58)	
Other	267/4395	1.00 (Reference)	0.75 (0.35, 1.59)	0.73 (0.37, 1.44)	0.68 (0.29, 1.62)	0.85 (0.31, 2.32)	
**Education**							0.353
less than 9th grade	1611/5784	1.00 (Reference)	0.77 (0.58, 1.02)	0.85 (0.63, 1.16)	0.76 (0.56, 1.03)	0.88 (0.55, 1.41)	
9-11th grade	1548/7577	1.00 (Reference)	0.75 (0.58, 0.97)	0.60 (0.47, 0.77)	0.69 (0.53, 0.90)	0.70 (0.52, 0.95)	
College graduate or above	1278/13386	1.00 (Reference)	0.70 (0.47, 1.06)	0.83 (0.55, 1.25)	0.62 (0.42, 0.91)	0.62 (0.40, 0.94)	
High school graduate/GED or equivalent	2099/12686	1.00 (Reference)	0.96 (0.70, 1.30)	1.13 (0.81, 1.58)	0.94 (0.68, 1.30)	0.79 (0.55, 1.14)	
Some college or AA degree	2006/16575	1.00 (Reference)	0.87 (0.67, 1.14)	0.89 (0.69, 1.15)	0.82 (0.62, 1.09)	0.65 (0.47, 0.90)	
**Income**							0.615
$ 0 to $ 19,999	3093/12829	1.00 (Reference)	0.79 (0.66, 0.96)	0.90 (0.73, 1.12)	0.84 (0.67, 1.04)	0.75 (0.55, 1.02)	
$20,000 to $44,999	3356/18656	1.00 (Reference)	0.82 (0.67, 1.01)	0.83 (0.67, 1.02)	0.72 (0.56, 0.91)	0.59 (0.45, 0.77)	

Characteristics	Case/N	Total DAC					*P* for interaction
		Quintile 1	Quintile 2	Quintile 3	Quintile 4	Quintile 5	
**(continued)**							
$45,000 to $74,999	1206/10832	1.00 (Reference)	0.86 (0.56, 1.33)	0.95 (0.65, 1.40)	0.89 (0.63, 1.27)	0.84 (0.58, 1.22)	
$75,000 to $99,999	746/9026	1.00 (Reference)	0.92 (0.53, 1.60)	0.62 (0.39, 0.98)	0.57 (0.36, 0.92)	0.61 (0.35, 1.09)	
$100,000 and Over	165/4723	1.00 (Reference)	1.23 (0.48, 3.15)	1.59 (0.60, 4.22)	1.49 (0.56, 3.93)	1.00 (0.28, 3.49)	
**Body mass index (kg/m^2)**							0.331
>25	2570/17134	1.00 (Reference)	0.75 (0.60, 0.94)	0.79 (0.63, 0.99)	0.74 (0.58, 0.96)	0.56 (0.41, 0.76)	
25 to 29	3065/19093	1.00 (Reference)	0.86 (0.69, 1.07)	0.91 (0.70, 1.18)	0.68 (0.53, 0.86)	0.70 (0.52, 0.96)	
≥30	2931/19839	1.00 (Reference)	0.90 (0.70, 1.18)	0.89 (0.72, 1.10)	0.94 (0.73, 1.21)	0.81 (0.59, 1.10)	
**Smoking status**							<0.001*
Never smoked	3443/30509	1.00 (Reference)	0.84 (0.63, 1.11)	0.88 (0.67, 1.15)	0.83 (0.63, 1.10)	0.79 (0.58, 1.06)	
Past smoker	3438/14074	1.00 (Reference)	0.83 (0.67, 1.01)	0.85 (0.69, 1.04)	0.76 (0.60, 0.97)	0.69 (0.53, 0.89)	
Current smoker	1685/11483	1.00 (Reference)	0.94 (0.74, 1.19)	0.98 (0.76, 1.27)	0.84 (0.65, 1.07)	0.63 (0.47, 0.84)	
**Drinking (g/day)**							0.435
Tertile 1	4484/18689	1.00 (Reference)	0.83 (0.67, 1.03)	0.81 (0.67, 0.98)	0.73 (0.60, 0.88)	0.64 (0.50, 0.83)	
Tertile 2	2005/18689	1.00 (Reference)	0.91 (0.73, 1.13)	0.94 (0.76, 1.18)	0.98 (0.80, 1.21)	0.78 (0.58, 1.05)	
Tertile 3	2077/18688	1.00 (Reference)	0.75 (0.53, 1.06)	0.88 (0.65, 1.19)	0.71 (0.50, 1.01)	0.75 (0.56, 1.01)	
**HEI-2015 score**							0.255
<70	7662/51043	1.00 (Reference)	0.84 (0.72, 0.98)	0.86 (0.74, 0.99)	0.77 (0.67, 0.89)	0.66 (0.56, 0.78)	
≥70	904/5023	1.00 (Reference)	0.95 (0.26, 3.46)	1.20 (0.38, 3.77)	1.01 (0.32, 3.22)	0.94 (0.29, 3.01)	
**Physical activity (METs-h/week)**							0.351
<5	2762/17438	1.00 (Reference)	0.71 (0.55, 0.93)	0.77 (0.63, 0.94)	0.67 (0.53, 0.85)	0.65 (0.51, 0.82)	
5 to 11	1316/13694	1.00 (Reference)	0.72 (0.52, 0.99)	1.02 (0.69, 1.51)	0.74 (0.52, 1.07)	0.59 (0.39, 0.88)	
≥12	4488/24934	1.00 (Reference)	0.94 (0.81, 1.08)	0.86 (0.74, 1.00)	0.84 (0.72, 1.00)	0.74 (0.60, 0.92)	

Abbreviations: HRs, hazard ratio; CIs, confidence intervals; DAC, dietary total antioxidant capacity; HEI-2015, Healthy Eating Index 2015; BMI, body mass index; CVD, cardiovascular disease. HR (95%CI) was estimated by weighted Cox regression analyses. Model was adjusted for, age, sex, race, education, family income, dietary energy intake, alcohol consumption per day, smoking status, physical activity, BMI, diabetes, hypertension, CVD, cancer, hyperlipidaemia, adherence to HEI-2015 score, and dietary supplement use except for the one that defined the group.

Supplementary Table 11 Association of dinner DAC and all-cause mortality by ten characteristics

Characteristics	Case/N	Dinner DAC					*P* for interaction
		Quintile 1	Quintile 2	Quintile 3	Quintile 4	Quintile 5	
**Age (years)**							<0.001*
age<65	2671/43027	1.00 (Reference)	0.93 (0.78, 1.12)	0.79 (0.63, 1.00)	0.84 (0.65, 1.08)	0.68 (0.46, 1.00)	
age≥65	5895/13039	1.00 (Reference)	0.99 (0.86, 1.13)	0.94 (0.81, 1.08)	0.92 (0.78, 1.08)	0.89 (0.71, 1.11)	
**Sex**							0.837
Men	4832/28133	1.00 (Reference)	0.97 (0.82, 1.15)	0.88 (0.74, 1.05)	0.85 (0.71, 1.02)	0.81 (0.63, 1.05)	
Women	3734/27933	1.00 (Reference)	0.90 (0.73, 1.10)	0.91 (0.75, 1.11)	0.91 (0.71, 1.16)	0.85 (0.63, 1.14)	
**Race/ethnicity**							0.831
Mexican American	1074/9314	1.00 (Reference)	1.30 (0.93, 1.80)	1.30 (0.85, 1.97)	1.13 (0.79, 1.61)	0.99 (0.55, 1.76)	
Non-Hispanic Black	1630/11411	1.00 (Reference)	0.98 (0.74, 1.30)	1.15 (0.89, 1.49)	1.04 (0.78, 1.38)	1.01 (0.72, 1.42)	
Non-Hispanic White	5261/26797	1.00 (Reference)	0.91 (0.78, 1.06)	0.83 (0.73, 0.94)	0.84 (0.70, 1.01)	0.78 (0.60, 1.00)	
Other Hispanic	334/4149	1.00 (Reference)	1.46 (0.92, 2.32)	2.74 (1.12, 6.69)	1.35 (0.80, 2.26)	2.32 (1.06, 5.08)	
Other	267/4395	1.00 (Reference)	0.87 (0.47, 1.62)	0.71 (0.34, 1.49)	0.74 (0.34, 1.64)	0.77 (0.26, 2.25)	
**Education**							0.312
less than 9th grade	1611/5784	1.00 (Reference)	1.21 (0.90, 1.63)	1.32 (0.94, 1.84)	1.23 (0.88, 1.71)	1.03 (0.61, 1.73)	
9-11th grade	1548/7577	1.00 (Reference)	0.99 (0.76, 1.29)	0.73 (0.55, 0.97)	0.65 (0.47, 0.89)	0.52 (0.35, 0.78)	
College graduate or above	1278/13386	1.00 (Reference)	0.96 (0.66, 1.39)	0.93 (0.62, 1.39)	0.95 (0.64, 1.42)	0.88 (0.55, 1.40)	
High school graduate/GED or equivalent	2099/12686	1.00 (Reference)	0.94 (0.72, 1.21)	0.82 (0.65, 1.03)	0.85 (0.63, 1.15)	0.68 (0.43, 1.08)	
Some college or AA degree	2006/16575	1.00 (Reference)	0.83 (0.62, 1.11)	0.98 (0.74, 1.31)	0.92 (0.67, 1.24)	1.09 (0.71, 1.68)	
**Income**							0.993
$ 0 to $ 19,999	3093/12829	1.00 (Reference)	1.07 (0.88, 1.30)	0.95 (0.80, 1.13)	0.97 (0.78, 1.20)	1.17 (0.82, 1.65)	
$20,000 to $44,999	3356/18656	1.00 (Reference)	0.88 (0.75, 1.04)	0.91 (0.76, 1.08)	0.77 (0.64, 0.94)	0.69 (0.53, 0.91)	

Characteristics	Case/N	Dinner DAC					*P* for interaction
		Quintile 1	Quintile 2	Quintile 3	Quintile 4	Quintile 5	
**(continued)**							
$45,000 to $74,999	1206/10832	1.00 (Reference)	1.04 (0.81, 1.34)	1.03 (0.74, 1.43)	1.12 (0.81, 1.56)	0.79 (0.54, 1.15)	
$75,000 to $99,999	746/9026	1.00 (Reference)	0.77 (0.49, 1.21)	0.67 (0.41, 1.11)	0.78 (0.46, 1.33)	0.77 (0.39, 1.52)	
$100,000 and Over	165/4723	1.00 (Reference)	1.08 (0.48, 2.42)	0.91 (0.46, 1.80)	1.62 (0.85, 3.10)	1.40 (0.62, 3.17)	
**Body mass index (kg/m^2)**							0.506
>25	2570/17134	1.00 (Reference)	1.08 (0.85, 1.36)	1.00 (0.80, 1.25)	1.04 (0.80, 1.37)	0.94 (0.68, 1.29)	
25 to 29	3065/19093	1.00 (Reference)	0.99 (0.80, 1.22)	0.91 (0.75, 1.11)	0.91 (0.71, 1.16)	0.83 (0.57, 1.22)	
≥30	2931/19839	1.00 (Reference)	0.80 (0.66, 0.96)	0.81 (0.64, 1.03)	0.76 (0.59, 0.99)	0.72 (0.52, 0.99)	
**Smoking status**							0.027*
Never smoked	3443/30509	1.00 (Reference)	0.92 (0.75, 1.14)	0.87 (0.69, 1.09)	0.95 (0.75, 1.21)	0.85 (0.63, 1.16)	
Past smoker	3438/14074	1.00 (Reference)	0.92 (0.75, 1.12)	0.94 (0.77, 1.16)	0.80 (0.64, 1.00)	0.77 (0.57, 1.05)	
Current smoker	1685/11483	1.00 (Reference)	1.00 (0.79, 1.28)	0.89 (0.68, 1.17)	0.92 (0.68, 1.23)	0.76 (0.43, 1.37)	
**Drinking (g/day)**							0.265
Tertile 1	4484/18689	1.00 (Reference)	0.96 (0.82, 1.13)	0.97 (0.82, 1.13)	0.89 (0.74, 1.07)	0.80 (0.62, 1.04)	
Tertile 2	2005/18689	1.00 (Reference)	1.05 (0.84, 1.32)	1.02 (0.84, 1.23)	1.03 (0.81, 1.31)	1.01 (0.70, 1.46)	
Tertile 3	2077/18688	1.00 (Reference)	0.84 (0.65, 1.07)	0.70 (0.54, 0.91)	0.77 (0.58, 1.03)	0.72 (0.49, 1.06)	
**HEI-2015 score**							0.087
<70	7662/51043	1.00 (Reference)	0.95 (0.84, 1.09)	0.93 (0.83, 1.04)	0.87 (0.75, 1.02)	0.81 (0.64, 1.03)	
≥70	904/5023	1.00 (Reference)	0.94 (0.62, 1.40)	0.74 (0.44, 1.24)	1.08 (0.70, 1.66)	0.88 (0.56, 1.37)	
**Physical activity (METs-h/week)**							0.911
<5	2762/17438	1.00 (Reference)	0.77 (0.64, 0.93)	0.79 (0.65, 0.95)	0.75 (0.60, 0.94)	0.68 (0.51, 0.90)	
5 to 11	1316/13694	1.00 (Reference)	0.89 (0.67, 1.19)	0.98 (0.74, 1.30)	1.02 (0.73, 1.42)	0.98 (0.61, 1.58)	
≥12	4488/24934	1.00 (Reference)	1.05 (0.90, 1.23)	0.94 (0.82, 1.08)	0.93 (0.79, 1.09)	0.92 (0.73, 1.17)	

Abbreviations: HRs, hazard ratio; CIs, confidence intervals; DAC, dietary total antioxidant capacity; HEI-2015, Healthy Eating Index 2015; BMI, body mass index; CVD, cardiovascular disease. HR (95%CI) was estimated by weighted Cox regression analyses. Model was adjusted for, age, sex, race, education, family income, dietary energy intake, alcohol consumption per day, smoking status, physical activity, BMI, diabetes, hypertension, CVD, cancer, hyperlipidaemia, adherence to HEI-2015 score, and dietary supplement use except for the one that defined the group.

Supplementary Table 12 Associations of Δ DAC and all-cause mortality by ten characteristics

Characteristics	Case/N	Δ DAC					*P* for interaction
		Quintile 1	Quintile 2	Quintile 3	Quintile 4	Quintile 5	
**Age (years)**							0.055
age<65	2671/43027	1.00 (Reference)	1.04 (0.75, 1.43)	0.90 (0.63, 1.29)	0.75 (0.53, 1.06)	0.61 (0.39, 0.95)	
age≥65	5895/13039	1.00 (Reference)	0.87 (0.77, 0.99)	0.93 (0.80, 1.09)	0.82 (0.69, 0.97)	0.78 (0.65, 0.94)	
**Sex**							0.491
Men	4832/28133	1.00 (Reference)	0.89 (0.73, 1.09)	0.95 (0.75, 1.19)	0.81 (0.63, 1.03)	0.71 (0.54, 0.94)	
Women	3734/27933	1.00 (Reference)	1.01 (0.84, 1.21)	1.00 (0.81, 1.24)	0.93 (0.77, 1.13)	0.91 (0.69, 1.20)	
**Race/ethnicity**							0.967
Mexican American	1074/9314	1.00 (Reference)	1.02 (0.68, 1.54)	1.37 (0.88, 2.12)	1.08 (0.64, 1.82)	0.83 (0.48, 1.44)	
Non-Hispanic Black	1630/11411	1.00 (Reference)	1.21 (0.93, 1.57)	1.36 (0.97, 1.92)	1.50 (1.08, 2.07)	1.53 (0.94, 2.49)	
Non-Hispanic White	5261/26797	1.00 (Reference)	0.86 (0.73, 1.01)	0.85 (0.71, 1.01)	0.72 (0.61, 0.85)	0.64 (0.50, 0.82)	
Other Hispanic	334/4149	1.00 (Reference)	2.39 (1.32, 4.31)	2.69 (1.13, 6.41)	3.77 (1.33, 10.63)	5.37 (1.84, 15.67)	
Other	267/4395	1.00 (Reference)	0.95 (0.55, 1.64)	0.92 (0.41, 2.08)	0.84 (0.38, 1.83)	0.75 (0.32, 1.73)	
**Education**							0.187
less than 9th grade	1611/5784	1.00 (Reference)	0.90 (0.66, 1.22)	1.12 (0.86, 1.45)	1.32 (0.93, 1.86)	0.77 (0.50, 1.18)	
9-11th grade	1548/7577	1.00 (Reference)	0.88 (0.68, 1.13)	0.92 (0.70, 1.22)	0.73 (0.54, 0.99)	0.67 (0.45, 1.01)	
College graduate or above	1278/13386	1.00 (Reference)	1.02 (0.74, 1.41)	1.19 (0.84, 1.68)	1.00 (0.68, 1.48)	0.80 (0.50, 1.28)	
High school graduate/GED or equivalent	2099/12686	1.00 (Reference)	0.82 (0.61, 1.11)	0.76 (0.55, 1.05)	0.69 (0.49, 0.98)	0.65 (0.39, 1.09)	
Some college or AA degree	2006/16575	1.00 (Reference)	0.99 (0.75, 1.33)	0.96 (0.70, 1.33)	0.77 (0.54, 1.09)	0.90 (0.58, 1.41)	
**Income**							0.047*
$ 0 to $ 19,999	3093/12829	1.00 (Reference)	0.98 (0.81, 1.18)	1.09 (0.87, 1.37)	1.09 (0.90, 1.33)	1.03 (0.76, 1.39)	
$20,000 to $44,999	3356/18656	1.00 (Reference)	1.00 (0.80, 1.25)	0.97 (0.80, 1.17)	0.82 (0.65, 1.04)	0.76 (0.56, 1.05)	

Characteristics	Case/N	Δ DAC					*P* for interaction
		Quintile 1	Quintile 2	Quintile 3	Quintile 4	Quintile 5	
**(continued)**							
$45,000 to $74,999	1206/10832	1.00 (Reference)	0.81 (0.62, 1.06)	0.79 (0.58, 1.06)	0.78 (0.56, 1.09)	0.74 (0.48, 1.16)	
$75,000 to $99,999	746/9026	1.00 (Reference)	0.87 (0.48, 1.57)	1.06 (0.53, 2.14)	0.65 (0.31, 1.35)	0.58 (0.27, 1.27)	
$100,000 and Over	165/4723	1.00 (Reference)	1.10 (0.45, 2.67)	0.95 (0.39, 2.30)	0.87 (0.38, 2.01)	0.67 (0.23, 1.95)	
**Body mass index (kg/m^2)**							0.635
>25	2570/17134	1.00 (Reference)	1.01 (0.80, 1.28)	1.09 (0.85, 1.39)	0.96 (0.73, 1.27)	1.07 (0.77, 1.49)	
25 to 29	3065/19093	1.00 (Reference)	0.82 (0.66, 1.01)	0.82 (0.64, 1.04)	0.78 (0.60, 1.00)	0.53 (0.39, 0.73)	
≥30	2931/19839	1.00 (Reference)	1.01 (0.80, 1.27)	1.01 (0.81, 1.26)	0.86 (0.65, 1.13)	0.88 (0.61, 1.26)	
**Smoking status**							0.922
Never smoked	3443/30509	1.00 (Reference)	0.86 (0.69, 1.07)	0.91 (0.72, 1.14)	0.84 (0.67, 1.06)	0.71 (0.54, 0.94)	
Past smoker	3438/14074	1.00 (Reference)	0.98 (0.82, 1.17)	1.09 (0.90, 1.33)	0.91 (0.73, 1.13)	0.88 (0.67, 1.16)	
Current smoker	1685/11483	1.00 (Reference)	0.79 (0.55, 1.15)	0.70 (0.48, 1.03)	0.62 (0.43, 0.90)	0.59 (0.36, 0.98)	
**Drinking (g/day)**							0.947
Tertile 1	4484/18689	1.00 (Reference)	0.92 (0.76, 1.13)	0.97 (0.80, 1.18)	0.86 (0.70, 1.06)	0.73 (0.56, 0.94)	
Tertile 2	2005/18689	1.00 (Reference)	0.86 (0.70, 1.06)	0.91 (0.72, 1.15)	0.78 (0.62, 0.98)	0.84 (0.60, 1.18)	
Tertile 3	2077/18688	1.00 (Reference)	0.99 (0.71, 1.38)	0.95 (0.67, 1.34)	0.83 (0.57, 1.22)	0.78 (0.48, 1.26)	
**HEI-2015 score**							0.177
<70	7662/51043	1.00 (Reference)	0.89 (0.77, 1.04)	0.92 (0.77, 1.08)	0.81 (0.69, 0.96)	0.71 (0.55, 0.91)	
≥70	904/5023	1.00 (Reference)	1.11 (0.79, 1.57)	1.32 (0.89, 1.96)	1.00 (0.69, 1.43)	1.27 (0.81, 1.99)	
**Physical activity (METs-h/week)**							0.296
<5	2762/17438	1.00 (Reference)	1.00 (0.80, 1.23)	1.02 (0.81, 1.28)	0.87 (0.68, 1.10)	0.79 (0.58, 1.07)	
5 to 11	1316/13694	1.00 (Reference)	1.09 (0.77, 1.53)	1.22 (0.79, 1.90)	1.08 (0.72, 1.64)	0.96 (0.55, 1.69)	
≥12	4488/24934	1.00 (Reference)	0.86 (0.74, 1.01)	0.89 (0.75, 1.06)	0.79 (0.66, 0.95)	0.76 (0.59, 0.98)	

Abbreviations: HRs, hazard ratio; CIs, confidence intervals; DAC, dietary total antioxidant capacity; HEI-2015, Healthy Eating Index 2015; BMI, body mass index; CVD, cardiovascular disease. HR (95%CI) was estimated by weighted Cox regression analyses. Model was adjusted for, age, sex, race, education, family income, dietary energy intake, alcohol consumption per day, smoking status, physical activity, BMI, diabetes, hypertension, CVD, cancer, hyperlipidaemia, adherence to HEI-2015 score, and dietary supplement use except for the one that defined the group.

Supplementary Table 13 Baseline characteristics of biochemical indicators, categorized by quintiles of total, dinner, and △ DACs

**Total DAC**	Variable	Quintile 1	Quintile 2	Quintile 3	Quintile 4	Quintile 5	*P* value
	Fasting glucose (mmol/L)	5.05±0.02	5.09±0.02	5.09±0.02	5.08±0.02	5.01±0.02	0.010
	Glycohemoglobin (%)	5.48±0.01	5.51±0.01	5.56±0.02	5.52±0.02	5.44±0.02	<0.001
	HOMA-IR	1.49±0.06	1.66±0.06	1.55±0.06	1.41±0.05	1.29±0.04	<0.001
	HOMA-IS	2.43±0.04	2.25±0.04	2.24±0.04	2.25±0.05	2.26±0.05	0.010
	Triglyceride-glucose index	8.62±0.01	8.63±0.01	8.62±0.01	8.60±0.01	8.54±0.01	<0.001
	HDL-cholesterol (mg/dL)	58.56±0.65	58.63±0.61	59.69±0.60	60.91±0.49	63.44±0.59	<0.001
**Dinner DAC**	Variable	Quintile 1	Quintile 2	Quintile 3	Quintile 4	Quintile 5	*P* value
	Fasting glucose (mmol/L)	5.08±0.02	5.08±0.02	5.07±0.02	5.06±0.02	5.03±0.02	0.310
	Glycohemoglobin (%)	5.53±0.01	5.54±0.01	5.49±0.01	5.49±0.02	5.47±0.02	<0.001
	HOMA-IR	1.50±0.05	1.49±0.05	1.55±0.06	1.48±0.05	1.35±0.05	0.030
	HOMA-IS	2.27±0.04	2.36±0.04	2.30±0.04	2.26±0.05	2.26±0.04	0.460
	Triglyceride-glucose index	8.66±0.01	8.61±0.01	8.59±0.01	8.59±0.01	8.56±0.01	<0.001
	HDL-cholesterol (mg/dL)	58.51±0.48	60.37±0.58	60.46±0.60	60.16±0.55	61.85±0.58	<0.001
**Δ DAC**	Variable	Quintile 1	Quintile 2	Quintile 3	Quintile 4	Quintile 5	*P* value
	Fasting glucose (mmol/L)	5.05±0.02	5.08±0.02	5.07±0.02	5.08±0.02	5.03±0.02	0.180
	Glycohemoglobin (%)	5.52±0.02	5.56±0.01	5.51±0.01	5.48±0.02	5.46±0.01	<0.001
	HOMA-IR	1.37±0.06	1.47±0.04	1.52±0.07	1.57±0.06	1.43±0.05	0.030
	HOMA-IS	2.22±0.05	2.26±0.05	2.41±0.04	2.29±0.04	2.26±0.04	0.003
	Triglyceride-glucose index	8.58±0.01	8.64±0.01	8.61±0.01	8.59±0.01	8.58±0.01	0.002
	HDL-cholesterol (mg/dL)	61.35±0.61	59.42±0.49	59.70±0.57	60.12±0.60	60.94±0.51	0.020

Abbreviations: DAC, dietary total antioxidant capacity. Continuous variables were adjusted for survey weights of NHANES. Categorical variables were unweighted. One-way ANOVA for continuous variables and chi-square test of independence test for categorical variables were performed.

Supplementary Table 14 Association between total DAC, dinner DAC, and △ DAC with biochemical indicators

Exposure	Outcome	β value (95% CI)	*P* value
Total DAC	Serum CRP	-0. 18 (-0.22, -0.14)	<0.001
	Glycohemoglobin	-0.02 (-0.04, -0.01)	<0.001
	Plasma fasting glucose	-0.03 (-0.08, 0.03)	0.391
	HOMA-IR	-0.01 (-0.04, 0.03)	0.750
	HOMA-IS	-0.01 (-0.03, 0.01)	0.398
	HDL-cholesterol	0.10 (-0.09, 0.30)	0.302
	Triglyceride-glucose index	0.00 (-0.02, 0.01)	0.605
Dinner DAC	Serum CRP	-0.12 (-0.16, -0.09)	<0.001
	Glycohemoglobin	-0.01 (-0.03, 0.01)	0.294
	Plasma fasting glucose	-0.04 (-0.11, 0.03)	0.255
	HOMA-IR	0.02 (-0.03, 0.06)	0.444
	HOMA-IS	-0.02 (-0.04, 0.01)	0.229
	HDL-cholesterol	-0.05 (-0.30, 0.19)	0.674
	Triglyceride-glucose index	-0.02 (-0.03, 0.00)	0.011
△ DAC	Serum CRP	-0.07 (-0.12, -0.03)	0.001
	Glycohemoglobin	-0.01 (-0.02, 0.00)	0.243
	Plasma fasting glucose	-0.03 (-0.08, 0.02)	0.178
	HOMA-IR	0.03 (0.00, 0.05)	0.037
	HOMA-IS	-0.03 (-0.05, 0.00)	0.019
	HDL-cholesterol	0.08 (-0.07, 0.23)	0.319
	Triglyceride-glucose index	0.00 (-0.01, 0.01)	0.456

Abbreviations: DAC, dietary total antioxidant capacity; CRP, C-reactive protein. Δ equals dinner DAC minus breakfast DAC.

Model was adjusted for, age, sex, race, education, family income, dietary energy intake, alcohol consumption per day, smoking status, physical activity, BMI, diabetes, hypertension, CVD, cancer, hyperlipidaemia, adherence to HEI-2015 score, and dietary supplement use.

Models for dinner DAC was further adjusted for breakfast DAC and lunch DAC.

Supplementary Table 15 Associations of all-cause mortality with DAC (calculated by median) across three meals

Exposure	Case/N	Model 1		Model 2		Model 3	
		HR (95% CI)	*P* value	HR (95% CI)	*P* value	HR (95% CI)	*P* value
**Total DAC**							
Continuous total DAC	8566/56066	0.90 (0.89, 0.92)	<0.001	0.94 (0.92, 0.96)	<0.001	0.95 (0.93, 0.98)	<0.001
Quintile 1	1210/11214	1.00 (Reference)		1.00 (Reference)		1.00 (Reference)	
Quintile 2	1635/11213	0.75 (0.68, 0.83)		0.80 (0.72, 0.89)		0.82 (0.74, 0.92)	
Quintile 3	1771/11213	0.77 (0.67, 0.89)		0.87 (0.75, 1.01)		0.88 (0.76, 1.03)	
Quintile 4	1952/11213	0.60 (0.53, 0.69)		0.72 (0.63, 0.82)		0.75 (0.65, 0.87)	
Quintile 5	1998/11213	0.51 (0.45, 0.57)	<0.001*	0.64 (0.56, 0.73)	<0.001*	0.70 (0.61, 0.80)	<0.001*
**Breakfast DAC**							
Continuous breakfast DAC	8566/56066	0.91 (0.88, 0.93)	<0.001	0.97 (0.94, 1.00)	0.032	1.00 (0.97, 1.03)	0.777
Quintile 1	954/11214	1.00 (Reference)		1.00 (Reference)		1.00 (Reference)	
Quintile 2	1479/11213	0.95 (0.81, 1.11)		0.92 (0.78, 1.09)		0.93 (0.80, 1.10)	
Quintile 3	1921/11213	0.87 (0.76, 0.99)		0.87 (0.76, 1.00)		0.88 (0.77, 1.02)	
Quintile 4	1917/11213	0.86 (0.74, 1.00)		0.95 (0.81, 1.13)		0.98 (0.83, 1.15)	
Quintile 5	2295/11213	0.72 (0.63, 0.83)	<0.001*	0.87 (0.75, 1.00)	0.136*	0.93 (0.80, 1.09)	0.754*
**Lunch DAC**							
Continuous lunch DAC	8566/56066	0.92 (0.89, 0.95)	<0.001	0.95 (0.93, 0.98)	0.004	0.96 (0.93, 0.99)	0.011
Quintile 1	1208/11214	1.00 (Reference)		1.00 (Reference)		1.00 (Reference)	
Quintile 2	1739/11213	1.12 (0.98, 1.27)		1.06 (0.92, 1.21)		1.02 (0.89, 1.17)	
Quintile 3	1827/11213	1.05 (0.93, 1.19)		1.03 (0.92, 1.17)		1.01 (0.89, 1.14)	
Quintile 4	1914/11213	1.00 (0.88, 1.13)		1.05 (0.92, 1.19)		1.01 (0.90, 1.15)	
Quintile 5	1878/11213	0.82 (0.72, 0.92)	<0.001*	0.91 (0.80, 1.04)	0.084*	0.92 (0.80, 1.05)	0.187*

**Exposure**	**Case/N**	**Model 1**		**Model 2**		**Model 3**	
		**HR (95% CI)**	***P* value**	**HR (95% CI)**	***P* value**	**HR (95% CI)**	***P* value**
**(continued)**							
**Dinner DAC**							
Continuous dinner DAC	8566/56066	0.88 (0.84, 0.91)	<0.001	0.93 (0.89, 0.97)	0.001	0.94 (0.89, 0.98)	0.003
Quintile 1	1537/11214	1.00 (Reference)		1.00 (Reference)		1.00 (Reference)	
Quintile 2	2043/11213	0.92 (0.83, 1.02)		0.90 (0.81, 1.00)		0.87 (0.78, 0.96)	
Quintile 3	1806/11213	0.86 (0.77, 0.96)		0.91 (0.81, 1.01)		0.85 (0.76, 0.95)	
Quintile 4	1526/11213	0.76 (0.67, 0.87)		0.84 (0.74, 0.96)		0.80 (0.70, 0.91)	
Quintile 5	1654/11213	0.68 (0.60, 0.77)	<0.001*	0.79 (0.70, 0.90)	<0.001*	0.77 (0.68, 0.87)	<0.001*
**Δ DAC**							
Continuous Δ DAC	8566/56066	1.00 (0.98, 1.02)	0.811	0.99 (0.97, 1.01)	0.203	0.98 (0.95, 1.00)	0.057
Quintile 1	2183/11214	1.00 (Reference)		1.00 (Reference)		1.00 (Reference)	
Quintile 2	1922/11213	1.13 (1.01, 1.26)		1.01 (0.91, 1.12)		0.96 (0.86, 1.07)	
Quintile 3	1778/11213	1.28 (1.15, 1.43)		1.08 (0.97, 1.21)		1.02 (0.90, 1.15)	
Quintile 4	1367/11213	1.11 (0.99, 1.24)		1.02 (0.92, 1.12)		0.96 (0.86, 1.06)	
Quintile 5	1316/11213	0.85 (0.75, 0.97)	0.067*	0.84 (0.74, 0.95)	0.018*	0.82 (0.72, 0.93)	0.003*

Abbreviations: HRs, hazard ratio; CIs, confidence intervals; DAC, dietary total antioxidant capacity; HEI-2015, Healthy Eating Index 2015; BMI, body mass index; CVD, cardiovascular disease.

* *P* for trend across the quintile of DAC. HR (95%CI) was estimated by weighted Cox regression analyses. Δ equals dinner DAC minus breakfast DAC.

Model 1 adjusted for, age, sex, and race.

Model 2 further adjusted for education, family income, dietary energy intake, alcohol consumption per day, smoking status, physical activity, and BMI.

Model 3 further adjusted for diabetes, hypertension, CVD, cancer, hyperlipidaemia, adherence to HEI-2015 score, and dietary supplement use.

Models for breakfast DAC, lunch DAC, and dinner DAC were further adjusted except for the one that defined the group.

Supplementary Table 16 Associations of CVD mortality with quintiles of DAC (calculated by median) across three meals

Exposure	Case/N	Model 1		Model 2		Model 3	
		HR (95% CI)	*P* value	HR (95% CI)	*P* value	HR (95% CI)	*P* value
**Total DAC**							
Continuous total DAC	2196/56066	0.90 (0.87, 0.93)	<0.001	0.94 (0.91, 0.98)	0.002	0.98 (0.94, 1.02)	0.265
Quintile 1	282/11214	1.00 (Reference)		1.00 (Reference)		1.00 (Reference)	
Quintile 2	416/11213	0.74 (0.58, 0.94)		0.75 (0.60, 0.94)		0.77 (0.61, 0.97)	
Quintile 3	457/11213	0.84 (0.60, 1.19)		0.92 (0.63, 1.33)		0.96 (0.66, 1.40)	
Quintile 4	537/11213	0.67 (0.51, 0.88)		0.79 (0.60, 1.03)		0.89 (0.67, 1.19)	
Quintile 5	504/11213	0.49 (0.38, 0.62)	<0.001*	0.63 (0.49, 0.81)	0.002*	0.78 (0.59, 1.02)	0.309*
**Breakfast DAC**							
Continuous breakfast DAC	2196/56066	0.88 (0.83, 0.93)	<0.001	0.95 (0.90, 1.01)	0.108	1.00 (0.94, 1.06)	0.954
Quintile 1	214/11214	1.00 (Reference)		1.00 (Reference)		1.00 (Reference)	
Quintile 2	368/11213	1.02 (0.75, 1.38)		0.92 (0.65, 1.30)		0.92 (0.66, 1.29)	
Quintile 3	507/11213	0.84 (0.65, 1.09)		0.76 (0.57, 1.01)		0.77 (0.58, 1.02)	
Quintile 4	500/11213	0.86 (0.66, 1.11)		0.88 (0.66, 1.16)		0.92 (0.69, 1.23)	
Quintile 5	607/11213	0.71 (0.56, 0.89)	<0.001*	0.81 (0.63, 1.04)	0.118*	0.92 (0.71, 1.20)	0.874*
**Lunch DAC**							
Continuous lunch DAC	2196/56066	0.94 (0.88, 0.99)	0.026	0.99 (0.94, 1.05)	0.822	1.00 (0.94, 1.07)	
Quintile 1	260/11214	1.00 (Reference)		1.00 (Reference)		1.00 (Reference)	
Quintile 2	451/11213	1.51 (1.12, 2.02)		1.37 (0.98, 1.92)		1.31 (0.95, 1.80)	
Quintile 3	514/11213	1.56 (1.23, 2.00)		1.50 (1.16, 1.93)		1.46 (1.15, 1.85)	
Quintile 4	495/11213	1.29 (1.01, 1.66)		1.35 (1.03, 1.76)		1.31 (1.01, 1.69)	
Quintile 5	476/11213	1.04 (0.80, 1.37)	0.136*	1.21 (0.91, 1.61)	0.638*	1.25 (0.95, 1.65)	0.337*

Exposure	Case/N	Model 1		Model 2		Model 3	
		HR (95% CI)	*P* value	HR (95% CI)	*P* value	HR (95% CI)	*P* value
**(continued)**							
**Dinner DAC**							
Continuous dinner DAC	2196/56066	0.85 (0.79, 0.90)	<0.001	0.90 (0.85, 0.96)	0.001	0.93 (0.87, 0.99)	0.025
Quintile 1	355/11214	1.00 (Reference)		1.00 (Reference)		1.00 (Reference)	
Quintile 2	548/11213	0.93 (0.76, 1.13)		0.86 (0.69, 1.06)		0.81 (0.65, 1.02)	
Quintile 3	478/11213	0.88 (0.70, 1.11)		0.87 (0.68, 1.11)		0.82 (0.64, 1.06)	
Quintile 4	376/11213	0.74 (0.58, 0.93)		0.77 (0.61, 0.98)		0.76 (0.60, 0.96)	
Quintile 5	439/11213	0.71 (0.57, 0.88)	<0.001*	0.80 (0.64, 1.00)	0.030*	0.84 (0.66, 1.06)	0.132*
**Δ DAC**							
Continuous Δ DAC	2196/56066	1.00 (0.97, 1.03)	0.992	0.98 (0.95, 1.02)	0.346	0.97 (0.93, 1.01)	0.097
Quintile 1	571/11214	1.00 (Reference)		1.00 (Reference)		1.00 (Reference)	
Quintile 2	506/11213	1.17 (0.97, 1.41)		1.03 (0.85, 1.24)		0.94 (0.78, 1.14)	
Quintile 3	447/11213	1.34 (1.07, 1.67)		1.11 (0.88, 1.39)		0.98 (0.77, 1.25)	
Quintile 4	339/11213	1.07 (0.86, 1.32)		0.95 (0.77, 1.18)		0.86 (0.69, 1.08)	
Quintile 5	333/11213	0.87 (0.71, 1.05)	0.223*	0.84 (0.69, 1.02)	0.084*	0.81 (0.67, 0.99)	0.026*

Abbreviations: HRs, hazard ratios; CIs, confidence intervals; DAC, dietary total antioxidant capacity; HEI-2015, Healthy Eating Index 2015; BMI, body mass index; CVD, cardiovascular disease.

* *P* for trend across the quintile of DAC. HR (95%CI) was estimated by weighted Cox regression analyses. Δ equals dinner DAC minus breakfast DAC.

Model 1 adjusted for, age, sex, and race.

Model 2 further adjusted for education, family income, dietary energy intake, alcohol consumption per day, smoking status, physical activity, and BMI.

Model 3 further adjusted for diabetes, hypertension, CVD, cancer, hyperlipidaemia, adherence to HEI-2015 score, and dietary supplement use.

Models for breakfast DAC, lunch DAC, and dinner DAC were further adjusted except for the one that defined the group.

Supplementary Table 17 Associations of cancer mortality with quintiles of DAC (calculated by median) across three meals

Exposure	Case/N	Model 1		Model 2		Model 3	
		HR (95% CI)	*P* value	HR (95% CI)	*P* value	HR (95% CI)	*P* value
**Total DAC**							
Continuous total DAC	1984/56066	0.92 (0.89, 0.96)	<0.001	0.95 (0.91, 1.00)	0.039	0.96 (0.92, 1.01)	0.132
Quintile 1	294/11214	1.00 (Reference)		1.00 (Reference)		1.00 (Reference)	
Quintile 2	409/11213	1.05 (0.82, 1.34)		1.10 (0.85, 1.42)		1.14 (0.87, 1.48)	
Quintile 3	417/11213	1.05 (0.82, 1.34)		1.16 (0.90, 1.50)		1.18 (0.90, 1.54)	
Quintile 4	431/11213	0.72 (0.57, 0.91)		0.82 (0.65, 1.04)		0.86 (0.66, 1.11)	
Quintile 5	433/11213	0.66 (0.53, 0.83)	<0.001*	0.79 (0.61, 1.02)	0.001*	0.83 (0.62, 1.12)	0.016*
**Breakfast DAC**							
Continuous breakfast DAC	1984/56066	0.90 (0.83, 0.96)	0.004	0.95 (0.88, 1.02)	0.137	0.96 (0.88, 1.04)	0.33
Quintile 1	266/11214	1.00 (Reference)		1.00 (Reference)		1.00 (Reference)	
Quintile 2	360/11213	0.95 (0.72, 1.26)		0.86 (0.65, 1.14)		0.87 (0.65, 1.15)	
Quintile 3	419/11213	0.79 (0.62, 1.02)		0.74 (0.57, 0.96)		0.75 (0.57, 0.97)	
Quintile 4	445/11213	0.86 (0.65, 1.13)		0.88 (0.67, 1.16)		0.91 (0.69, 1.21)	
Quintile 5	494/11213	0.71 (0.54, 0.92)	0.002*	0.78 (0.61, 1.00)	0.132*	0.82 (0.63, 1.08)	0.366*
**Lunch DAC**							
Continuous lunch DAC	1984/56066	0.95 (0.89, 1.01)	0.104	0.97 (0.91, 1.04)	0.44	0.98 (0.92, 1.05)	0.599
Quintile 1	329/11214	1.00 (Reference)		1.00 (Reference)		1.00 (Reference)	
Quintile 2	380/11213	1.03 (0.80, 1.33)		0.92 (0.71, 1.19)		0.90 (0.70, 1.18)	
Quintile 3	399/11213	0.97 (0.77, 1.22)		0.88 (0.70, 1.12)		0.86 (0.67, 1.10)	
Quintile 4	463/11213	1.12 (0.86, 1.45)		1.08 (0.83, 1.40)		1.07 (0.82, 1.39)	
Quintile 5	413/11213	0.88 (0.67, 1.15)	0.482*	0.91 (0.69, 1.21)	0.976*	0.94 (0.70, 1.27)	0.797*

Exposure	Case/N	Model 1		Model 2		Model 3	
		HR (95% CI)	*P* value	HR (95% CI)	*P* value	HR (95% CI)	*P* value
**(continued)**							
**Dinner DAC**							
Continuous dinner DAC	1984/56066	0.93 (0.85, 1.02)	0.134	0.98 (0.89, 1.07)	0.642	0.98 (0.90, 1.08)	0.753
Quintile 1	383/11214	1.00 (Reference)		1.00 (Reference)		1.00 (Reference)	
Quintile 2	469/11213	1.07 (0.86, 1.33)		1.00 (0.79, 1.27)		0.97 (0.76, 1.23)	
Quintile 3	386/11213	0.90 (0.71, 1.15)		0.90 (0.71, 1.15)		0.86 (0.67, 1.10)	
Quintile 4	367/11213	0.87 (0.66, 1.14)		0.91 (0.70, 1.19)		0.88 (0.68, 1.13)	
Quintile 5	379/11213	0.78 (0.60, 1.02)	0.016*	0.86 (0.65, 1.13)	0.186*	0.84 (0.64, 1.09)	0.133*
**Δ DAC**							
Continuous Δ DAC	1984/56066	1.03 (0.97, 1.08)	0.349	1.02 (0.96, 1.08)	0.522	1.02 (0.95, 1.09)	0.594
Quintile 1	484/11214	1.00 (Reference)		1.00 (Reference)		1.00 (Reference)	
Quintile 2	431/11213	1.04 (0.84, 1.28)		0.94 (0.77, 1.16)		0.91 (0.73, 1.14)	
Quintile 3	389/11213	1.16 (0.93, 1.44)		0.97 (0.77, 1.21)		0.93 (0.72, 1.19)	
Quintile 4	348/11213	1.15 (0.92, 1.45)		1.05 (0.83, 1.32)		1.00 (0.77, 1.30)	
Quintile 5	332/11213	0.95 (0.72, 1.25)	0.931*	0.93 (0.71, 1.23)	0.885*	0.91 (0.69, 1.22)	0.762*

Abbreviations: HRs, hazard ratios; CIs, confidence intervals; DAC, dietary total antioxidant capacity; HEI-2015, Healthy Eating Index 2015; BMI, body mass index; CVD, cardiovascular disease.

* *P* for trend across the quintile of DAC. HR (95%CI) was estimated by weighted Cox regression analyses. Δ equals dinner DAC minus breakfast DAC.

Model 1 adjusted for, age, sex, and race.

Model 2 further adjusted for education, family income, dietary energy intake, alcohol consumption per day, smoking status, physical activity, and BMI.

Model 3 further adjusted for diabetes, hypertension, CVD, cancer, hyperlipidaemia, adherence to HEI-2015 score, and dietary supplement use.

Models for breakfast DAC, lunch DAC, and dinner DAC were further adjusted except for the one that defined the group.

Supplementary Table 18 All-cause, CVD, and cancer mortality risk by quintiles of total or dinner DAC plus midnight

Exposure	All-cause mortality		CVD mortality		Cancer mortality	
	HR (95% CI)	*P* value	HR (95% CI)	*P* value	HR (95% CI)	*P* value
**Total DAC plus midnight DAC [a]**						
Continuous total DAC plus midnight	0.98 (0.96, 0.99)	0.007	0.97 (0.94, 1.01)	0.105	0.98 (0.95, 1.02)	0.327
Q1 (<1.79)	1.00 (Reference)		1.00 (Reference)		1.00 (Reference)	
Q2 (1.79 to 2.73)	0.86 (0.74, 1.00)		0.87 (0.65, 1.18)		1.00 (0.76, 1.32)	
Q3 (2.73 to 3.97)	0.89 (0.77, 1.03)		0.86 (0.63, 1.18)		1.01 (0.79, 1.29)	
Q4 (3.97 to 6.00)	0.80 (0.69, 0.93)		0.91 (0.70, 1.18)		0.84 (0.64, 1.09)	
Q5 (> 6.00)	0.77 (0.66, 0.90)	0.002*	0.69 (0.53, 0.90)	0.028*	0.83 (0.64, 1.08)	0.068*
**Dinner DAC plus midnight DAC**						
Continuous dinner DAC plus midnight	0.97 (0.94, 1.00)	0.037	0.92 (0.88, 0.98)	0.004	1.01 (0.94, 1.07)	0.851
Q1 (<0.19)	1.00 (Reference)		1.00 (Reference)		1.00 (Reference)	
Q2 (0.19 to 0.56)	0.94 (0.84, 1.05)		0.90 (0.72, 1.12)		1.05 (0.83, 1.32)	
Q3 (0.56 to 1.09)	0.86 (0.77, 0.96)		0.83 (0.66, 1.05)		0.77 (0.61, 0.96)	
Q4 (1.09 to 2.12)	0.83 (0.73, 0.94)		0.86 (0.69, 1.06)		0.86 (0.67, 1.11)	
Q5 (> 2.12)	0.86 (0.75, 0.99)	0.002*	0.75 (0.59, 0.96)	0.014*	1.01 (0.76, 1.34)	0.490*
**Midnight DAC**						
Continuous midnight DAC	1.05 (0.97, 1.13)	0.22	0.90 (0.79, 1.02)	0.106	1.00 (0.86, 1.15)	0.96
Q1 (<-0.09)	1.00 (Reference)		1.00 (Reference)		1.00 (Reference)	
Q2 (-0.09 to 0.02)	0.87 (0.78, 0.98)		0.90 (0.71, 1.15)		0.83 (0.67, 1.05)	
Q3 (0.02 to 0.11)	1.03 (0.92, 1.15)		0.94 (0.72, 1.22)		0.98 (0.79, 1.21)	
Q4 (0.11 to 0.23)	1.05 (0.91, 1.22)		1.08 (0.79, 1.49)		0.93 (0.69, 1.25)	
Q5 (> 0.23)	1.02 (0.89, 1.16)	0.296*	0.90 (0.68, 1.20)	0.801*	0.84 (0.65, 1.09)	0.366*

Abbreviations: HRs, hazard ratios; CIs, confidence intervals; DAC, dietary total antioxidant capacity; HEI-2015, Healthy Eating Index 2015; BMI, body mass index; CVD, cardiovascular disease.

[a] DAC intake from midnight snack after dinner.

HR (95%CI) was estimated by weighted Cox regression analyses.

Model was adjusted for age, sex and race, education, family income, dietary energy intake, alcohol consumption per day, smoking status, physical activity, BMI, diabetes, hypertension, CVD, cancer, hyperlipidaemia, adherence to HEI-2015 score, and dietary supplement use. .

Models for total DAC plus midnight DAC, dinner DAC plus midnight DAC, and midnight DAC were further adjusted except for the one that defined the group.

Supplementary Table 19 All-cause, CVD, and cancer mortality by DAC quintiles plus DAC from snack, coffee, and/or tea

Exposure	All-cause mortality			CVD mortality			Cancer mortality		
	Case/N	HR(95% CI)	*P* value	Case/N	HR(95% CI)	*P* value	Case/N	HR(95% CI)	*P* value
**Total DAC (including DAC from snack, coffee and tea)**									
Continuous total DAC	8566/56066	0.99 (0.99, 1.00)	0.001	2196/56066	1.00 (0.98, 1.01)	0.399	1984/56066	1.00 (0.99, 1.01)	0.722
Quintile 1	1044/11214	1.00 (Reference)		262/11214	1.00 (Reference)		216/11214	1.00 (Reference)	
Quintile 2	1470/11213	0.82 (0.70, 0.96)		377/11213	0.87 (0.66, 1.15)		321/11213	0.88 (0.63, 1.22)	
Quintile 3	1903/11213	0.80 (0.70, 0.90)		467/11213	0.75 (0.59, 0.96)		460/11213	0.93 (0.70, 1.24)	
Quintile 4	1972/11213	0.64 (0.56, 0.73)		521/11213	0.72 (0.55, 0.93)		443/11213	0.72 (0.53, 0.98)	
Quintile 5	2177/11213	0.66 (0.57, 0.76)	<0.001*	569/11213	0.72 (0.55, 0.94)	0.008*	544/11213	0.84 (0.61, 1.15)	0.177*
**Total DAC (including DAC from snack)**									
Continuous total DAC	8566/56066	0.97 (0.95, 0.98)	<0.001	2196/56066	0.97 (0.94, 1.00)	0.023	1984/56066	0.98 (0.95, 1.02)	0.336
Quintile 1	1251/11214	1.00 (Reference)		318/11214	1.00 (Reference)		309/11214	1.00 (Reference)	
Quintile 2	1797/11213	0.79 (0.70, 0.91)		465/11213	0.69 (0.52, 0.91)		412/11213	0.85 (0.66, 1.08)	
Quintile 3	1913/11213	0.80 (0.71, 0.90)		495/11213	0.75 (0.58, 0.97)		454/11213	0.96 (0.75, 1.23)	
Quintile 4	1875/11213	0.69 (0.60, 0.79)		497/11213	0.67 (0.52, 0.85)		411/11213	0.80 (0.60, 1.08)	
Quintile 5	1730/11213	0.65 (0.55, 0.77)	<0.001*	421/11213	0.61 (0.45, 0.81)	0.004*	398/11213	0.87 (0.60, 1.27)	0.501*
**Total DAC (including DAC from coffee)**									
Continuous total DAC	8566/56066	0.99 (0.98, 0.99)	<0.001	2196/56066	0.98 (0.97, 1.00)	0.023	1984/56066	1.00 (0.99, 1.01)	0.432
Quintile 1	1010/11214	1.00 (Reference)		240/11214	1.00 (Reference)		235/11214	1.00 (Reference)	
Quintile 2	1388/11213	0.82 (0.71, 0.95)		390/11213	0.97 (0.74, 1.28)		258/11213	0.72 (0.50, 1.06)	
Quintile 3	1835/11213	0.73 (0.64, 0.84)		450/11213	0.63 (0.49, 0.80)		426/11213	0.83 (0.64, 1.09)	

Exposure	All-cause mortality			CVD mortality			Cancer mortality		
	Case/N	HR(95% CI)	*P* value	Case/N	HR(95% CI)	*P* value	Case/N	HR(95% CI)	*P* value
**(continued)**									
Quintile 4	2124/11213	0.65 (0.57, 0.75)		540/11213	0.68 (0.52, 0.88)		521/11213	0.74 (0.55, 0.99)	
Quintile 5	2209/11213	0.61 (0.52, 0.71)	<0.001*	576/11213	0.62 (0.48, 0.81)	<0.001*	544/11213	0.72 (0.54, 0.95)	0.056*
**Total DAC (including DAC from tea)**									
Continuous total DAC	8566/56066	0.99 (0.98, 1.00)	0.017	2196/56066	1.01 (0.99, 1.03)	0.286	1984/56066	0.99 (0.97, 1.01)	0.219
Quintile 1	1308/11214	1.00 (Reference)		298/11214	1.00 (Reference)		315/11214	1.00 (Reference)	
Quintile 2	1693/11213	0.83 (0.73, 0.95)		421/11213	0.84 (0.64, 1.11)		424/11213	1.09 (0.84, 1.42)	
Quintile 3	1764/11213	0.77 (0.67, 0.88)		465/11213	0.85 (0.65, 1.12)		386/11213	0.87 (0.67, 1.15)	
Quintile 4	1889/11213	0.77 (0.66, 0.89)		503/11213	0.90 (0.66, 1.24)		442/11213	0.94 (0.74, 1.19)	
Quintile 5	1912/11213	0.75 (0.66, 0.86)	<0.001*	509/11213	0.86 (0.66, 1.12)	0.582*	417/11213	0.85 (0.63, 1.13)	0.108*
**Breakfast DAC (including DAC from breakfast snack, coffee and tea)**									
Continuous breakfast DAC	8566/56066	0.99 (0.98, 1.00)	0.002	2196/56066	0.99 (0.97, 1.00)	0.052	1984/56066	1.00 (0.99, 1.02)	0.74
Quintile 1	939/11214	1.00 (Reference)		215/11214	1.00 (Reference)		255/11214	1.00 (Reference)	
Quintile 2	1466/11213	0.88 (0.76, 1.01)		381/11213	0.79 (0.58, 1.06)		330/11213	1.06 (0.80, 1.40)	
Quintile 3	1732/11213	0.93 (0.79, 1.09)		436/11213	0.82 (0.62, 1.08)		355/11213	0.96 (0.72, 1.28)	
Quintile 4	2254/11213	0.83 (0.72, 0.96)		631/11213	0.82 (0.64, 1.07)		483/11213	0.91 (0.67, 1.23)	
Quintile 5	2175/11213	0.76 (0.65, 0.88)	<0.001*	533/11213	0.67 (0.50, 0.89)	0.010*	561/11213	0.96 (0.71, 1.30)	0.537*
**Breakfast DAC (including DAC from breakfast snack)**									
Continuous breakfast DAC	8566/56066	1.00 (0.98, 1.02)	0.849	2196/56066	0.99 (0.95, 1.03)	0.571	1984/56066	0.99 (0.94, 1.05)	0.787

Exposure	All-cause mortality			CVD mortality			Cancer mortality		
	Case/N	HR(95% CI)	*P* value	Case/N	HR(95% CI)	*P* value	Case/N	HR(95% CI)	*P* value
**(continued)**									
Quintile 1	908/11214	1.00 (Reference)		194/11214	1.00 (Reference)		233/11214	1.00 (Reference)	
Quintile 2	1498/11213	1.03 (0.89, 1.20)		381/11213	1.02 (0.72, 1.45)		392/11213	1.26 (0.94, 1.67)	
Quintile 3	1956/11213	0.97 (0.85, 1.11)		514/11213	0.89 (0.65, 1.21)		426/11213	1.06 (0.79, 1.41)	
Quintile 4	1965/11213	1.06 (0.92, 1.22)		535/11213	1.08 (0.79, 1.48)		454/11213	1.23 (0.93, 1.64)	
Quintile 5	2239/11213	1.03 (0.88, 1.20)	0.589*	572/11213	0.95 (0.72, 1.26)	0.972*	479/11213	1.11 (0.79, 1.55)	0.765*
**Breakfast DAC (including DAC from breakfast coffee)**									
Continuous breakfast DAC	8566/56066	0.99 (0.98, 1.00)	0.007	2196/56066	0.98 (0.97, 1.00)	0.048	1984/56066	1.00 (0.98, 1.02)	0.979
Quintile 1	947/11214	1.00 (Reference)		220/11214	1.00 (Reference)		258/11214	1.00 (Reference)	
Quintile 2	1450/11213	0.84 (0.72, 0.98)		383/11213	0.76 (0.57, 0.99)		324/11213	0.90 (0.66, 1.22)	
Quintile 3	1676/11213	0.90 (0.78, 1.04)		427/11213	0.82 (0.65, 1.04)		340/11213	0.76 (0.56, 1.01)	
Quintile 4	2255/11213	0.80 (0.69, 0.93)		607/11213	0.73 (0.57, 0.93)		497/11213	0.81 (0.58, 1.13)	
Quintile 5	2238/11213	0.76 (0.65, 0.89)	<0.001*	559/11213	0.68 (0.52, 0.89)	0.012*	565/11213	0.83 (0.62, 1.12)	0.305*
**Breakfast DAC (including DAC from breakfast tea)**									
Continuous breakfast DAC	8566/56066	1.00 (0.98, 1.02)	0.974	2196/56066	1.01 (0.97, 1.04)	0.792	1984/56066	0.98 (0.94, 1.03)	0.467
Quintile 1	932/11214	1.00 (Reference)		197/11214	1.00 (Reference)		255/11214	1.00 (Reference)	
Quintile 2	1511/11213	0.88 (0.76, 1.02)		375/11213	0.94 (0.66, 1.32)		386/11213	0.93 (0.68, 1.27)	
Quintile 3	2007/11213	0.85 (0.74, 0.98)		543/11213	0.86 (0.65, 1.13)		443/11213	0.80 (0.60, 1.09)	
Quintile 4	1996/11213	0.91 (0.79, 1.06)		542/11213	0.98 (0.74, 1.29)		453/11213	0.86 (0.63, 1.18)	
Quintile 5	2120/11213	0.93 (0.80, 1.08)	0.852*	539/11213	0.95 (0.73, 1.22)	0.968*	447/11213	0.82 (0.59, 1.13)	0.201*

Exposure	All-cause mortality			CVD mortality			Cancer mortality		
	Case/N	HR(95% CI)	*P* value	Case/N	HR(95% CI)	*P* value	Case/N	HR(95% CI)	*P* value
**(continued)**									
**Lunch DAC (including DAC from lunch snack, coffee and tea)**									
Continuous lunch DAC	8566/56066	0.99 (0.98, 1.00)	0.127	2196/56066	1.01 (0.98, 1.04)	0.542	1984/56066	1.00 (0.97, 1.03)	0.835
Quintile 1	1195/11214	1.00 (Reference)		273/11214	1.00 (Reference)		302/11214	1.00 (Reference)	
Quintile 2	1781/11213	1.01 (0.89, 1.15)		473/11213	1.14 (0.91, 1.42)		403/11213	0.94 (0.72, 1.22)	
Quintile 3	1858/11213	0.96 (0.84, 1.09)		492/11213	1.19 (0.95, 1.49)		417/11213	0.93 (0.71, 1.23)	
Quintile 4	1833/11213	0.95 (0.82, 1.10)		444/11213	1.07 (0.83, 1.38)		426/11213	1.02 (0.77, 1.35)	
Quintile 5	1899/11213	0.93 (0.81, 1.07)	0.168*	514/11213	1.17 (0.92, 1.50)	0.462*	436/11213	0.94 (0.70, 1.26)	0.928*
**Lunch DAC (including DAC from lunch snack)**									
Continuous lunch DAC	8566/56066	0.98 (0.96, 0.99)	0.014	2196/56066	1.01 (0.97, 1.04)	0.744	1984/56066	0.99 (0.95, 1.04)	0.658
Quintile 1	1241/11214	1.00 (Reference)		277/11214	1.00 (Reference)		321/11214	1.00 (Reference)	
Quintile 2	1837/11213	1.07 (0.97, 1.18)		480/11213	1.22 (0.97, 1.54)		417/11213	0.96 (0.73, 1.26)	
Quintile 3	1927/11213	1.07 (0.95, 1.19)		535/11213	1.29 (1.02, 1.63)		418/11213	0.94 (0.71, 1.26)	
Quintile 4	1945/11213	1.04 (0.93, 1.17)		490/11213	1.17 (0.90, 1.53)		477/11213	1.12 (0.85, 1.47)	
Quintile 5	1616/11213	0.96 (0.86, 1.07)	0.272*	414/11213	1.08 (0.81, 1.44)	0.939*	351/11213	0.83 (0.60, 1.15)	0.563*
**Lunch DAC (including DAC from lunch coffee)**									
Continuous lunch DAC	8566/56066	0.98 (0.96, 1.00)	0.087	2196/56066	1.00 (0.96, 1.03)	0.812	1984/56066	1.01 (0.98, 1.04)	0.455
Quintile 1	1207/11214	1.00 (Reference)		268/11214	1.00 (Reference)		318/11214	1.00 (Reference)	

Exposure	All-cause mortality			CVD mortality			Cancer mortality		
	Case/N	HR(95% CI)	*P* value	Case/N	HR(95% CI)	*P* value	Case/N	HR(95% CI)	*P* value
**(continued)**									
Quintile 2	1740/11213	0.94 (0.84, 1.07)		458/11213	1.08 (0.81, 1.44)		371/11213	0.96 (0.75, 1.23)	
Quintile 3	1782/11213	0.93 (0.83, 1.06)		508/11213	1.25 (0.98, 1.59)		391/11213	0.88 (0.68, 1.15)	
Quintile 4	1928/11213	0.96 (0.84, 1.10)		496/11213	1.10 (0.84, 1.43)		458/11213	1.10 (0.85, 1.42)	
Quintile 5	1909/11213	0.91 (0.79, 1.05)	0.338*	466/11213	1.09 (0.84, 1.41)	0.656*	446/11213	1.04 (0.79, 1.37)	0.410*
**Lunch DAC (including DAC from lunch tea)**									
Continuous lunch DAC	8566/56066	0.98 (0.97, 1.00)	0.037	2196/56066	1.01 (0.98, 1.05)	0.437	1984/56066	0.99 (0.95, 1.02)	0.467
Quintile 1	1243/11214	1.00 (Reference)		274/11214	1.00 (Reference)		331/11214	1.00 (Reference)	
Quintile 2	1788/11213	0.96 (0.84, 1.08)		461/11213	1.11 (0.84, 1.45)		406/11213	0.95 (0.74, 1.23)	
Quintile 3	1800/11213	0.94 (0.83, 1.07)		501/11213	1.23 (0.97, 1.55)		393/11213	0.89 (0.69, 1.15)	
Quintile 4	1894/11213	0.94 (0.82, 1.07)		484/11213	1.02 (0.79, 1.31)		454/11213	1.07 (0.82, 1.40)	
Quintile 5	1841/11213	0.90 (0.78, 1.02)	0.117*	476/11213	1.13 (0.90, 1.43)	0.680*	400/11213	0.92 (0.68, 1.26)	0.929*
**Dinner DAC (including DAC from dinner snack, coffee and tea)**									
Continuous dinner DAC	8566/56066	0.99 (0.98, 1.00)	0.21	2196/56066	1.01 (0.98, 1.04)	0.379	1984/56066	1.00 (0.98, 1.02)	0.713
Quintile 1	1476/11214	1.00 (Reference)		336/11214	1.00 (Reference)		386/11214	1.00 (Reference)	
Quintile 2	1715/11213	0.92 (0.80, 1.05)		464/11213	1.11 (0.82, 1.51)		380/11213	0.83 (0.64, 1.07)	
Quintile 3	1648/11213	0.87 (0.75, 1.01)		410/11213	0.95 (0.68, 1.31)		365/11213	0.79 (0.62, 1.00)	
Quintile 4	1668/11213	0.84 (0.73, 0.97)		462/11213	1.16 (0.85, 1.59)		365/11213	0.71 (0.55, 0.91)	
Quintile 5	2059/11213	0.88 (0.78, 0.99)	0.020*	524/11213	1.04 (0.79, 1.36)	0.743*	488/11213	0.87 (0.69, 1.11)	0.218*

Exposure	All-cause mortality			CVD mortality			Cancer mortality		
	Case/N	HR(95% CI)	*P* value	Case/N	HR(95% CI)	*P* value	Case/N	HR(95% CI)	*P* value
**(continued)**									
**Dinner DAC (including DAC from dinner snack)**									
Continuous dinner DAC	8566/56066	0.97 (0.95, 0.99)	0.002	2196/56066	0.97 (0.93, 1.00)	0.075	1984/56066	0.99 (0.95, 1.03)	0.545
Quintile 1	1514/11214	1.00 (Reference)		359/11214	1.00 (Reference)		393/11214	1.00 (Reference)	
Quintile 2	1922/11213	0.94 (0.82, 1.07)		500/11213	1.09 (0.79, 1.50)		436/11213	0.85 (0.68, 1.06)	
Quintile 3	1714/11213	0.81 (0.71, 0.93)		457/11213	0.87 (0.64, 1.18)		378/11213	0.80 (0.62, 1.04)	
Quintile 4	1789/11213	0.84 (0.73, 0.96)		477/11213	1.05 (0.80, 1.38)		386/11213	0.70 (0.55, 0.90)	
Quintile 5	1627/11213	0.84 (0.74, 0.96)	0.001*	403/11213	0.93 (0.73, 1.19)	0.433*	391/11213	0.90 (0.71, 1.14)	0.200*
**Dinner DAC (including DAC from dinner coffee)**									
Continuous dinner DAC	8566/56066	0.98 (0.97, 1.00)	0.023	2196/56066	0.99 (0.96, 1.02)	0.351	1984/56066	1.01 (0.98, 1.04)	0.592
Quintile 1	1470/11214	1.00 (Reference)		337/11214	1.00 (Reference)		366/11214	1.00 (Reference)	
Quintile 2	2009/11213	0.94 (0.83, 1.06)		528/11213	0.93 (0.72, 1.18)		468/11213	1.04 (0.81, 1.34)	
Quintile 3	1717/11213	0.87 (0.78, 0.98)		454/11213	0.94 (0.73, 1.20)		360/11213	0.79 (0.62, 1.01)	
Quintile 4	1499/11213	0.82 (0.72, 0.94)		393/11213	0.96 (0.75, 1.23)		343/11213	0.83 (0.62, 1.10)	
Quintile 5	1871/11213	0.86 (0.76, 0.98)	0.003*	484/11213	0.92 (0.72, 1.18)	0.675*	447/11213	0.95 (0.74, 1.23)	0.318*
**Dinner DAC (including DAC from dinner tea)**									
Continuous dinner DAC	8566/56066	0.99 (0.98, 1.01)	0.395	2196/56066	1.02 (0.98, 1.05)	0.305	1984/56066	1.00 (0.98, 1.02)	0.926
Quintile 1	1426/11214	1.00 (Reference)		332/11214	1.00 (Reference)		353/11214	1.00 (Reference)	
Quintile 2	1968/11213	0.95 (0.85, 1.07)		519/11213	1.00 (0.78, 1.28)		453/11213	1.03 (0.79, 1.33)	

Exposure	All-cause mortality			CVD mortality			Cancer mortality		
	Case/N	HR(95% CI)	*P* value	Case/N	HR(95% CI)	*P* value	Case/N	HR(95% CI)	*P* value
**(continued)**									
Quintile 3	1575/11213	0.87 (0.76, 0.99)		391/11213	0.88 (0.67, 1.16)		340/11213	0.83 (0.65, 1.06)	
Quintile 4	1511/11213	0.85 (0.72, 0.99)		394/11213	1.03 (0.73, 1.45)		349/11213	0.82 (0.64, 1.06)	
Quintile 5	2086/11213	0.91 (0.80, 1.03)	0.070*	560/11213	1.02 (0.81, 1.28)	0.753*	489/11213	1.00 (0.78, 1.27)	0.568*
**△DAC (including △DAC from snack, coffee and tea)**									
Continuous △DAC	8566/56066	1.01 (1.00, 1.01)	0.073	2196/56066	1.01 (1.00, 1.03)	0.131	1984/56066	1.00 (0.98, 1.01)	0.665
Quintile 1	2079/11214	1.00 (Reference)		525/11214	1.00 (Reference)		531/11214	1.00 (Reference)	
Quintile 2	2077/11213	1.16 (1.06, 1.27)		537/11213	1.13 (0.92, 1.39)		435/11213	1.04 (0.85, 1.27)	
Quintile 3	1688/11213	1.18 (1.06, 1.31)		424/11213	1.15 (0.90, 1.45)		390/11213	1.13 (0.89, 1.42)	
Quintile 4	1315/11213	1.17 (1.06, 1.31)		332/11213	1.16 (0.96, 1.42)		298/11213	1.10 (0.84, 1.43)	
Quintile 5	1407/11213	1.07 (0.97, 1.18)	0.072*	378/11213	1.20 (0.98, 1.47)	0.049*	330/11213	0.95 (0.75, 1.21)	0.985*
**△DAC (including △DAC from snack)**									
Continuous △DAC	8566/56066	0.99 (0.97, 1.00)	0.024	2196/56066	0.99 (0.96, 1.01)	0.225	1984/56066	1.00 (0.97, 1.03)	0.933
Quintile 1	2044/11214	1.00 (Reference)		518/11214	1.00 (Reference)		486/11214	1.00 (Reference)	
Quintile 2	1938/11213	0.97 (0.85, 1.10)		500/11213	1.05 (0.85, 1.30)		418/11213	0.67 (0.53, 0.84)	
Quintile 3	1724/11213	0.96 (0.85, 1.07)		436/11213	0.98 (0.78, 1.24)		395/11213	0.86 (0.65, 1.13)	
Quintile 4	1438/11213	0.84 (0.75, 0.95)		391/11213	0.94 (0.75, 1.18)		313/11213	0.67 (0.51, 0.88)	
Quintile 5	1422/11213	0.88 (0.78, 0.98)	0.003*	351/11213	0.93 (0.75, 1.15)	0.223*	372/11213	0.88 (0.67, 1.15)	0.331*
**△DAC (including △DAC from coffee)**									
Continuous △DAC	8566/56066	1.01 (1.00, 1.02)	0.021	2196/56066	1.01 (1.00, 1.03)	0.08	1984/56066	1.00 (0.98, 1.02)	0.821

Exposure	All-cause mortality			CVD mortality			Cancer mortality		
	Case/N	HR(95% CI)	*P* value	Case/N	HR(95% CI)	*P* value	Case/N	HR(95% CI)	*P* value
**(continued)**									
Quintile 1	2190/11214	1.00 (Reference)		552/11214	1.00 (Reference)		567/11214	1.00 (Reference)	
Quintile 2	2139/11213	1.10 (1.00, 1.21)		562/11213	1.07 (0.89, 1.29)		434/11213	0.98 (0.80, 1.21)	
Quintile 3	1681/11213	1.23 (1.09, 1.40)		421/11213	1.27 (1.00, 1.61)		377/11213	1.08 (0.87, 1.34)	
Quintile 4	1370/11213	1.17 (1.04, 1.32)		328/11213	1.12 (0.88, 1.44)		315/11213	0.99 (0.78, 1.24)	
Quintile 5	1186/11213	1.14 (0.99, 1.31)	0.013*	333/11213	1.32 (1.07, 1.61)	0.009*	291/11213	1.14 (0.84, 1.54)	0.483*
**△DAC (including △DAC from tea)**									
Continuous △DAC	8566/56066	1.00 (0.99, 1.02)	0.736	2196/56066	1.03 (0.99, 1.07)	0.206	1984/56066	0.99 (0.97, 1.02)	0.612
Quintile 1	1911/11214	1.00 (Reference)		495/11214	1.00 (Reference)		437/11214	1.00 (Reference)	
Quintile 2	1899/11213	0.96 (0.85, 1.08)		495/11213	0.96 (0.78, 1.18)		412/11213	0.83 (0.66, 1.05)	
Quintile 3	1635/11213	0.97 (0.85, 1.10)		391/11213	0.92 (0.74, 1.15)		380/11213	0.91 (0.70, 1.18)	
Quintile 4	1291/11213	0.91 (0.80, 1.03)		320/11213	0.90 (0.73, 1.10)		309/11213	0.89 (0.67, 1.19)	
Quintile 5	1830/11213	0.89 (0.79, 0.99)	0.034*	495/11213	0.93 (0.79, 1.11)	0.403*	446/11213	0.96 (0.75, 1.22)	0.996*

Abbreviations: HRs, hazard ratio; CIs, confidence intervals; DAC, dietary total antioxidant capacity; HEI-2015, Healthy Eating Index 2015; BMI, body mass index; CVD, cardiovascular disease.

* *P* for trend across the quintile of DAC. HR (95%CI) was estimated by weighted Cox regression analyses. Δ equals dinner DAC minus breakfast DAC.

Model was adjusted for, age, sex, race, education, family income, dietary energy intake, alcohol consumption per day, smoking status, physical activity, BMI, diabetes, hypertension, CVD, cancer, hyperlipidaemia, adherence to HEI-2015 score, and dietary supplement use.

Models for breakfast DAC, lunch DAC, and dinner DAC were further adjusted except for the one that defined the group.

Models for DAC including coffee were further adjusted for caffeine intake.

Supplementary Table 20 Associations of all-cause, CVD, and cancer mortality with DAC using non-imputation data

Exposure	All-cause mortality		CVD mortality		Cancer mortality	
	HR (95% CI)	*P* value	HR (95% CI)	*P* value	HR (95% CI)	*P* value
**Total DAC**						
Continuous total DAC	0.98 (0.95, 1.01)	0.128	1.01 (0.97, 1.06)	0.508	0.99 (0.94, 1.05)	0.734
Quintile 1	1.00 (Reference)		1.00 (Reference)		1.00 (Reference)	
Quintile 2	0.83 (0.67, 1.03)		0.68 (0.42, 1.12)		1.34 (0.84, 2.13)	
Quintile 3	0.90 (0.69, 1.16)		0.89 (0.55, 1.43)		1.28 (0.76, 2.16)	
Quintile 4	0.83 (0.64, 1.08)		0.99 (0.63, 1.56)		1.01 (0.66, 1.54)	
Quintile 5	0.80 (0.62, 1.03)	0.194*	0.82 (0.53, 1.27)	0.966*	0.97 (0.57, 1.66)	0.418*
**Breakfast DAC**						
Continuous breakfast DAC	1.02 (0.99, 1.06)	0.25	1.04 (0.98, 1.11)	0.161	1.01 (0.93, 1.09)	0.87
Quintile 1	1.00 (Reference)		1.00 (Reference)		1.00 (Reference)	
Quintile 2	0.83 (0.60, 1.14)		0.87 (0.41, 1.82)		1.02 (0.60, 1.73)	
Quintile 3	0.79 (0.60, 1.05)		0.95 (0.55, 1.65)		0.79 (0.49, 1.27)	
Quintile 4	0.91 (0.66, 1.25)		1.07 (0.63, 1.82)		0.80 (0.48, 1.30)	
Quintile 5	0.99 (0.71, 1.39)	0.570*	1.20 (0.68, 2.13)	0.173*	0.93 (0.55, 1.59)	0.620*
**Lunch DAC**						
Continuous lunch DAC	0.99 (0.96, 1.02)	0.393	1.06 (1.00, 1.12)	0.073	0.98 (0.91, 1.05)	0.569
Quintile 1	1.00 (Reference)		1.00 (Reference)		1.00 (Reference)	
Quintile 2	1.04 (0.83, 1.31)		1.35 (0.69, 2.66)		1.20 (0.78, 1.85)	
Quintile 3	0.99 (0.77, 1.26)		1.45 (0.88, 2.39)		1.15 (0.76, 1.73)	
Quintile 4	1.07 (0.85, 1.35)		1.32 (0.85, 2.04)		0.90 (0.55, 1.45)	
Quintile 5	1.04 (0.82, 1.32)	0.722*	1.53 (0.91, 2.55)	0.146*	1.14 (0.73, 1.76)	0.864*

Exposure	All-cause mortality		CVD mortality		Cancer mortality	
	HR (95% CI)	*P* value	HR (95% CI)	*P* value	HR (95% CI)	*P* value
**(continued)**						
**Dinner DAC**						
Continuous dinner DAC	0.96 (0.89, 1.03)	0.22	0.90 (0.80, 1.02)	0.095	1.03 (0.92, 1.15)	0.602
Quintile 1	1.00 (Reference)		1.00 (Reference)		1.00 (Reference)	
Quintile 2	0.73 (0.58, 0.91)		0.69 (0.42, 1.15)		0.69 (0.46, 1.05)	
Quintile 3	0.75 (0.60, 0.94)		0.80 (0.48, 1.34)		0.68 (0.42, 1.09)	
Quintile 4	0.73 (0.57, 0.93)		0.73 (0.45, 1.20)		0.89 (0.61, 1.31)	
Quintile 5	0.71 (0.54, 0.93)	0.041*	0.58 (0.34, 0.97)	0.085*	0.84 (0.53, 1.34)	0.953*
**Δ DAC**						
Continuous Δ DAC	0.97 (0.94, 1.00)	0.059	0.94 (0.89, 0.98)	0.009	1.02 (0.94, 1.10)	0.66
Quintile 1	1.00 (Reference)		1.00 (Reference)		1.00 (Reference)	
Quintile 2	0.92 (0.77, 1.11)		1.18 (0.78, 1.76)		0.69 (0.47, 1.00)	
Quintile 3	0.97 (0.74, 1.26)		0.92 (0.61, 1.38)		0.81 (0.49, 1.33)	
Quintile 4	0.83 (0.69, 1.01)		0.64 (0.40, 1.03)		0.99 (0.65, 1.51)	
Quintile 5	0.73 (0.57, 0.93)	0.005*	0.76 (0.49, 1.18)	0.033*	0.82 (0.53, 1.27)	0.886*

Abbreviations: HRs, hazard ratio; CIs, confidence intervals; DAC, dietary total antioxidant capacity; HEI-2015, Healthy Eating Index 2015; BMI, body mass index; CVD, cardiovascular disease.

* *P* for trend across the quintile of DAC. HR (95%CI) was estimated by weighted Cox regression analyses. Δ equals dinner DAC minus breakfast DAC.

Model was adjusted for, age, sex, race, education, family income, dietary energy intake, alcohol consumption per day, smoking status, physical activity, BMI, diabetes, hypertension, CVD, cancer, hyperlipidaemia, adherence to HEI-2015 score, and dietary supplement use.

Models for breakfast DAC, lunch DAC, and dinner DAC were further adjusted except for the one that defined the group.
